# Transcriptomic Differences Between Two *Fusarium oxysporum* Formae Speciales During Cucumber Infection

**DOI:** 10.3390/jof12070540

**Published:** 2026-07-22

**Authors:** Ernest Nailevich Komissarov, Alfred Onele Obinna, Inna Alexandrovna Abdeeva, Mariya Vladimirovna Mokryakova, Sergey Alexandrovich Bruskin, Shamil Zavdatovich Validov

**Affiliations:** 1 Laboratory of Molecular Genetics and Microbiology Methods, Kazan Scientific Center of the Russian Academy of Sciences, 420111 Kazan, Russia; donjay.ao@gmail.com (A.O.O.); sh.validov@knc.ru (S.Z.V.); 2 Vavilov Institute of General Genetics, Russian Academy of Sciences, 119991 Moscow, Russia; insaz@vigg.ru (I.A.A.); mokryakova@vigg.ru (M.V.M.); brouskin@vigg.ru (S.A.B.); 3 Moscow Center for Advanced Studies, 123592 Moscow, Russia

**Keywords:** *Fusarium oxysporum*, *radicis-lycopersici*, *radicis-cucumerinum*, root rot, transcriptome, accessory chromosomes, SIX genes

## Abstract

*Fusarium oxysporum* f. sp. *radicis-cucumerinum* (*Forc*) V03-2g and *Fusarium oxysporum* f. sp. *radicis-lycopersici* (*Forl*) ZUM2407 both cause foot and root rot in cucumber, but differ in host range. *Forc* V03-2g possesses Secreted in Xylem (SIX) effector genes, whereas *Forl* ZUM2407 does not, raising questions about their distinct infection strategies on this host. Using comparative transcriptomic analysis (in cucumber at 7 and 14 days post-inoculation (dpi) and in tomato at 2 dpi) we show that *Forl* ZUM2407 induces a delayed defense response in cucumber compared to *Forc* V03-2g. In turn, *Forc* V03-2g rapidly activates accessory chromosome effectors on cucumber, while *Forl* ZUM2407 initially deploys core chromosome genes, activating distinct from *Forc* V03-2g accessory genes only by 14 dpi. Thereby, *Forc* V03-2g and *Forl* ZUM2407 use distinct accessory gene repertoires (unique to each strain) and distinct core gene transcription strategies to infect the same host.

## 1. Introduction

The *Fusarium oxysporum* species complex (FOSC) comprises a diverse group of soil-borne fungi that include both non-pathogenic (saprotrophic or endophytic) and pathogenic strains characterized by a variable karyotype and the absence of a sexual stage [1]. Pathogenic members are responsible for vascular wilt and foot and root rot diseases in a wide range of economically important crops, leading to substantial agricultural losses worldwide [2]. A defining feature of pathogenic FOSC strains is their narrow host specificity, with individual strains typically infecting only one or a limited group of closely related plant species [3]. Strains sharing a common host range are classified into formae speciales (ff. spp.; singular f. sp.), an extra-taxonomic grouping [3].

Notably, f.sp. are polyphyletic, such that strains assigned to the same f.sp. may be more distantly related to one another than to non-pathogenic isolates within the FOSC [4]. This pattern supports the view that host specificity and pathogenicity are largely determined by horizontally acquired genetic material rather than by vertical inheritance within a single lineage [5]. Horizontal gene or chromosome transfer is thought to occur via parasexual processes, including mitotic recombination and the exchange of accessory chromosomes [6].

The genome of the FOSC is organized into eleven conserved core chromosomes, which are vertically inherited and encode essential cellular functions, and a variable accessory genome characterized by high repeat content, low gene density, and conditional dispensability [5,7,8,9]. Accessory chromosomes commonly carry genes associated with pathogenicity, and their role in virulence has been demonstrated, where the transfer of specific accessory chromosomes of *Fusarium oxysporum* (*Fo*) f. sp. *lycopersici* (*Fol*) and *Fo* f. sp. *radicis-cucumerinum* (*Forc*) led to virulence acquisition by non-pathogenic *Fo* strains [7,8].

In many ff.spp. causing vascular wilt, the accessory genome exhibits a characteristic structure enriched in repetitive sequences and mobile genetic elements, including miniature impala elements (MIMPs), and is marked by a relatively low gene density [9]. This compartment contains genes encoding Secreted in Xylem (SIX) proteins, as well as other small secreted proteins lacking annotated functional domains and frequently associated with MIMPs [9]. These effectors contribute to pathogenicity and are widely distributed among strains causing vascular wilt and such foot and root rot pathogen like *Forc* [10].

The repertoire of such effector genes has been used as a molecular basis for distinguishing ff.spp. and for resolving phylogenetic relationships among strains within individual f.sp. [11]. However, *Fo* f. sp. *radicis-lycopersici* (*Forl*) represents an exception to this pattern. Unlike *Fol*, which infects tomato, and *Fo* f. sp. *cucumerinum* (*Foc*) and *Forc*, which infect cucumber, *Forl* does not cluster with these ff.spp. and is characterized by a comparatively low abundance of candidate effector genes [11,12].

In our previous study, we performed a comparative pathogenicity and genomic analysis of two strains representing different ff.spp., *Forc* V03-2g and *Forl* ZUM2407, both capable of infecting cucumber [12]. On cucumber, *Forc* V03-2g induces severe foot and root rot symptoms, whereas *Forl* ZUM2407 causes milder symptoms in sterile sand by 14 dpi. On tomato, *Forl* ZUM2407 is highly aggressive and induces severe foot and root rot symptoms by 7 dpi under the same conditions, while *Forc* V03-2g shows no pathogenicity on tomato. These phenotypic differences were also reflected at the genomic level. The *Forc* V03-2g genome exhibited a virulence-associated accessory genome comparable in structure and composition to that of vascular wilt pathogens. In contrast, the *Forl* ZUM2407 genome contained no SIX genes, and had a low content of MIMPs and candidate effector genes [9,12]. Moreover, these strains shared only one candidate effector gene homolog from accessory genome, which was highly expressed in cucumber only in *Forc* V03-2g [12]. These findings raise the question of how phylogenetically and genomically distinct strains causing foot and root rot are able to infect the same host species.

In the present study, we analyze transcriptomic data obtained during interactions between *Forc* V03-2g and *Forl* ZUM2407 strains and their host plants, cucumber and tomato, to investigate the functional differences suggested by the pathogenicity and genomic analysis. These results not only corroborate our previous genomic observations but also provide new insights into the infection strategy and biological characteristics of the *Forl* ZUM2407 strain, which appears to differ substantially from well-characterized vascular wilt pathogens.

## 2. Materials and Methods

### 2.1. Fungal Strains and Plant Varieties

*Fusarium oxysporum* f.sp. *radicis-cucumerinum* (*Forc*) V03-2g used in this study was isolated from infected greenhouse cucumber plants in the All-Russian Research Institute of Agricultural Microbiology (Saint-Petersburg-Pushkin, Russia). *Fusarium oxysporum* f.sp. *radicis-lycopersici* (*Forl*) ZUM2407 used in this study was isolated from infected tomato plants by the DLO Research Institute for Plant Protection (Wageningen, The Netherlands). The pathogenic strains *Forc* V03-2g and *Forl* ZUM2407, which cause foot and root rot in cucumber (*Cucumis sativus* L.) and tomato (*Solanum lycopersicum* L.) [13], respectively, were provided by the laboratory of Molecular Genetics and Microbiology Methods.

The cucumber variety “Palchik” (Volzhskiy sad, Republic of Tatarstan, Russia) and tomato variety “Belyi Naliv” (Agrofirm POISK, Moscow, Russia), which are susceptible to foot and root rot caused by *Forc* V03-2g and *Forl* ZUM2407, respectively, used in this study were purchased from registered seed growers.

### 2.2. Plant and Fungi Growth Conditions and Plant Inoculation

The plant inoculation was performed under laboratory conditions as described in our previous work [12]. Briefly, tomato and cucumber seeds were sterilized as described by Simons et al. [14]. After sterilization, cucumber and tomato seeds were placed in a Petri dish moist chamber and incubated at 25 °C for pre-germination. Fungal spores were prepared from a 5-day-old culture of Forl ZUM2407 and Forc V03-2g grown in potato dextrose medium (PDA) [(potato broth—200 g/L), dextrose—2% (*m/v*)] and filtered through sterilized cotton wool. Spores were adjusted to 10^6^ spores/mL using a hemocytometer. Pre-germinated seeds were placed in the spore suspension for 30 min. Tomato seeds after inoculation were placed in a Petri dish moist chamber. Cucumber seeds were sown in plastic pots containing sterile sand amended with plant nutrient solution [15]. Cucumber plants were cultivated for 7 and 14 days, and tomato plants for 2 days, corresponding to early mostly asymptomatic (2–7 dpi) and later symptomatic (14 dpi) stages of infection. After cultivation, the cucumber plants were removed from the pot and washed with running tap water. After that, the disease index was calculated based on damping off, foot and root rot symptoms using a scale from 0 to 4 [12], and a 4 cm section centered at the root collar was excised for RNA isolation (pathogen localization) (Figure S1). Eight cucumber plants per replicate were collected. Whole tomato seedlings were used without excising, with 25 plants per replicate (Figure S2). For each biological replicate, total RNA was extracted from pooled plant material. In total, three biological replicates were collected for each sample. The disease index between groups was analyzed using a one-way ANOVA test (*p* < 0.05).

### 2.3. RNA Isolation and Sequencing

Total RNA was extracted from cucumber and tomato tissues (collected as described in Section 2.2) using the RNeasy Plant Mini Kit (Qiagen, Hilden, Germany) according to the manufacturer’s instructions, including an on-column DNase I treatment to remove residual genomic DNA. RNA concentration was measured using a Qubit fluorometer (Thermo Fisher Scientific, Waltham, MA, USA), and RNA integrity was assessed on an Agilent 2100 Bioanalyzer (Agilent Technologies, Santa Clara, CA, USA). All samples showed high integrity (RIN ≥ 7.0) and were used for library preparation.

Poly(A)-enriched sequencing libraries were prepared from 2 µg of total RNA per sample using the KAPA mRNA HyperPrep Kit (Roche, Basel, Switzerland) following the manufacturer’s protocol. Library size distribution was verified on an Agilent 2100 Bioanalyzer and concentrations were quantified by fluorometry.

Paired-end sequencing (2 × 150 bp) was performed on an Illumina NextSeq 1000 platform (Illumina, San Diego, CA, USA) at the “Genomics, Proteomics, Metabolomics” Core Facility of the Lopukhin Federal Research and Clinical Center of Physical-Chemical Medicine of the Federal Medical Biological Agency (Moscow, Russia). Three biological replicates were sequenced for each condition (cucumber and tomato infected and control samples at each time point). Raw sequencing reads were deposited in the NCBI Sequence Read Archive under BioProject accession number PRJNA1475220.

### 2.4. The Analysis of Differentially Expressed Genes

#### 2.4.1. Alignment and Assembly

For the analysis of both plant and fungal transcriptomes, raw sequencing reads were first assessed for quality using FastQC v. 0.12.1, and adapter sequences and low-quality bases were trimmed using Trimmomatic v. 0.40 [16]. The resulting high-quality reads were then aligned to the reference genomes using HISAT2 v. 2.2.1 [17]. For the cucumber and tomato samples, reads were aligned to the *Cucumis sativus* 9930_V3 (accession number: GCF_000004075.3) and *Solanum lycopersicum* SLM_r2.1 (accession number: GCF_036512215.1) genome assemblies, respectively. For the fungal samples, reads were aligned to the *Forc* V03-2g (accession number: GCA_048164945.1) and *Forl* ZUM2407 (accession number: GCA_048165035.1) genome assemblies obtained from the National Center for Biotechnology Information (NCBI) database (https://www.ncbi.nlm.nih.gov, accessed on 25 September 2025). To assess differential expression of core secreted genes between *Forc* V03-2g and *Forl* ZUM2407, reads were aligned to the eleven core chromosomes of the *Forc* V03-2g genome [12]. To minimize mapping bias due to sequence divergence between the two strains, we restricted our downstream analysis to genes that were present in both genomes with at least 95% nucleotide identity and 95% coverage, as confirmed by BLASTn v.2.16.0. Transcriptome assembly were performed using StringTie v. 2.2.3 [18]. The abundance of transcripts was quantified using the featureCounts tool v. 2.1.1 [19].

#### 2.4.2. Differential Expression Analysis

Differential expression analysis was carried out using the DESeq2 package v. 1.50.2 [20]. To account for potential compositional bias due to variation in fungal read proportions, we initially compared two models: one including fungal read percentage as a covariate, and one without. Since the covariate was strongly collinear with the group effect under conditions of active disease progression, its inclusion led to overcorrection that masked genuine group-specific signals. We therefore excluded the covariate from the final model. For each contrast, differentially expressed genes were defined as those with |log2 fold change| > 1, adjusted *p*-value < 0.01, and base mean > 50.

#### 2.4.3. Validation of Key Expression Patterns

To validate the biological relevance of key genes obtained by DESeq2 analysis without covariate, and to exclude the possibility that their expression changes are driven solely by compositional bias caused by differing fungal read percentages, we performed an independent, organism-specific normalization using normalized counts from DESeq2. For plant transcripts, we used a set of three housekeeping genes (β-tubulin, actin-7 and EF1-α). For fungal transcripts, we used β-tubulin, actin and TEF1-α. Both sets of reference genes were selected for high stability across all samples (CV < 25%, pairwise Pearson correlation > 0.9 for each set). For each sample and each organism separately, a reference value was calculated as the geometric mean of the three corresponding housekeeping genes. The ratio of each target gene (plant or fungal) to its organism-specific housekeeping reference was then used to evaluate relative expression, thereby normalizing for variation in total RNA pool size independently for each interacting partner. Correlation between individual fungal accessory gene ex-pression and the pooled plant immune response was calculated using the geometric mean of the plant immune gene set normalized to housekeeping genes (immune response index).

#### 2.4.4. Functional Annotation and Pathway Analysis

For the functional annotation of transcribed genes, the coding sequences were extracted using AGAT v. 1.4.0, translated into protein sequences using TransDecoder v. 5.5.0, and annotated using the eggNOG-mapper tool v. 2.1.8 (database v. 5.0.2) [21]. Secreted proteins were predicted using DeepSig v. 1.2.5 [22]. Overrepresentation of GO terms and KEGG pathways among plant differentially expressed genes was assessed in clusterProfiler v. 4.14.0 [23], using the eggNOG-derived term-to-gene maps. Canonical KEGG pathway diagrams were rendered with Pathview v. 1.46.0 [24] using ortholog-based reference maps for four defense-related pathways, namely plant–pathogen interaction (ko04626), plant hormone signal transduction (ko04075), MAPK signaling pathway plant (ko04016), and phenylpropanoid biosynthesis (ko00940). Plant genes were assigned to immune-related families using eggNOG mapper protein family domains. Visualization of differentially expressed genes were performed using R packages ggplot2 v.4.0.3 and TomicsVis v. 2.0.0 [25].

### 2.5. Relative RNA Quantification Analysis in Forc V03-2g and Forl ZUM2407

Relative RNA quantification (RQ) analysis in *Forc* V03-2g and *Forl* ZUM2407 was performed using in planta assays, as described in Section 2.2, to confirm fungal RNA-seq data trends. For this purpose, cucumber plants of the variety “Palchik” and tomato plants of the variety “Beliy Naliv” were inoculated with *Forc* V03-2g and *Forl* ZUM2407. For RNA isolation a 4 cm section centered at the root collar was excised at 7 and 14 dpi for tomato and cucumber plants. The whole seedlings of both plants were used at 2 dpi. The RQ values were evaluated at 2, 7, and 14 days post-inoculation as described in our previous work [12]. The β-tubulin gene was used as a reference for RT-qPCR normalization [26]. Its stability was initially supported by transcriptomic data, although it was not independently validated by qPCR across all time points. Thereby the RT-qPCR data are interpreted primarily in terms of expression trends rather than absolute quantitative comparisons. Statistical differences in relative gene expression between host plants were analyzed using a one-way ANOVA test (*p* < 0.05). Primers were designed using CloneManager v.9 (Table S1).

## 3. Results

### 3.1. Colonization Dynamics Assessed by Fungal RNA Read Abundance

To examine the molecular basis of pathogenesis in *Forc* V03-2g and *Forl* ZUM2407, sampling was performed at two time points of cucumber infection: at 14 days post-inoculation (dpi), coinciding with onset of clearly visible foot and root rot symptoms for both strains, and at 7 dpi as the middle of incubation period and mostly asymptomatic stage. For the additional data of potential virulence genes usage in another host, we used the tomato early asymptomatic infection stage at 2 dpi. Although the sampling time points differ between the two host plants, the tomato-derived data provide an interesting perspective that connects to the development of Forl ZUM2407 and Forc V03-2g on cucumbers.

**Figure 1:**
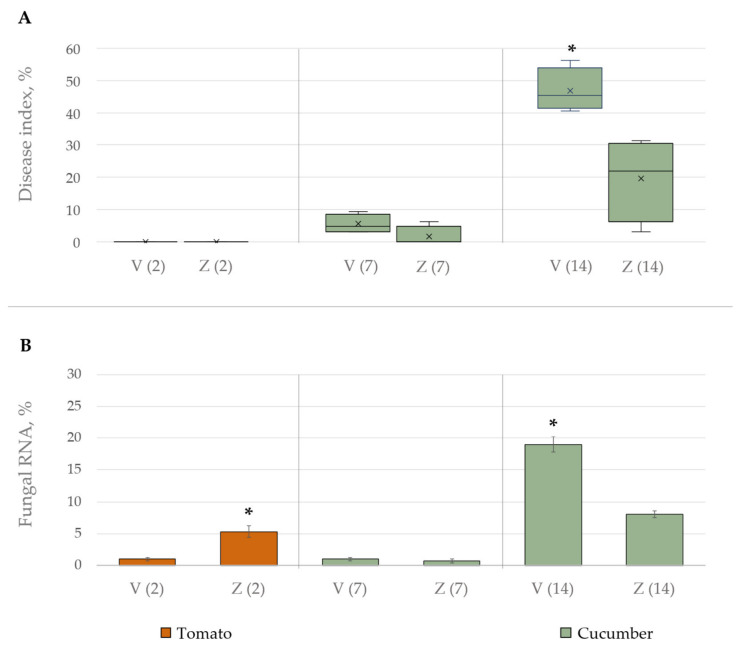
(**A**) Disease index of tomato and cucumber samples before RNA extraction. (**B**) Fungal colonization quantified as the proportion of fungal RNA-seq reads relative to the total number of reads in infected plants. Abbreviations: V—*Forc* V03-2g; Z—*Forl* ZUM2407; numbers in brackets (2, 7 and 14) indicate time points (dpi). Asterisks indicate statistically significant differences between V and Z at the indicated time points (ANOVA, *p* < 0.05).

Each RNA library generated approximately 25–38 million paired-end reads. In control plant samples, 91–95% of reads were mapped to the plant reference genomes. Additional sequencing and mapping statistics are summarized in Table S2. PCA variance and MA plots are shown in Figures S3 and S4a–S4e. Disease index of inoculated plants used for transcriptome analysis is represented in Figure 1A. Fungal biomass within plant tissues was estimated using the proportion of fungal RNA-seq reads relative to the total number of reads and is illustrated in Figure 1B.

In cucumber at 7 dpi, fungal read proportions were similar between the two strains, reaching 1.0 ± 0.3% for *Forc* V03-2g and 0.7 ± 0.3% for *Forl* ZUM2407. By 14 dpi, the proportion of fungal reads increased substantially in *Forc* V03-2g samples to 19.0 ± 1.2%, whereas *Forl* ZUM2407 reached 8.0 ± 0.6%. These data are consistent with the disease index, which at 7 dpi was 5.5 ± 2.6% for *Forc* V03-2g and 1.6 ± 2.7% for *Forl* ZUM2407, reaching 46.9 ± 5.8% and 19.5 ± 11.1% by 14 dpi, respectively. In tomato at 2 dpi, *Forl* ZUM2407 accounted for 5.3 ± 0.9% of total reads, whereas *Forc* V03-2g represented just 1.0 ± 0.2%. Symptoms of foot and root rot were not detected in tomato at 2 dpi, but slight growth retardation was observed in samples infected with *Forl* ZUM2407 (Figure S2).

### 3.2. Differential Gene Expression in Cucumber Following Inoculation with Forc V03-2g and Forl ZUM2407

**Figure 2:**
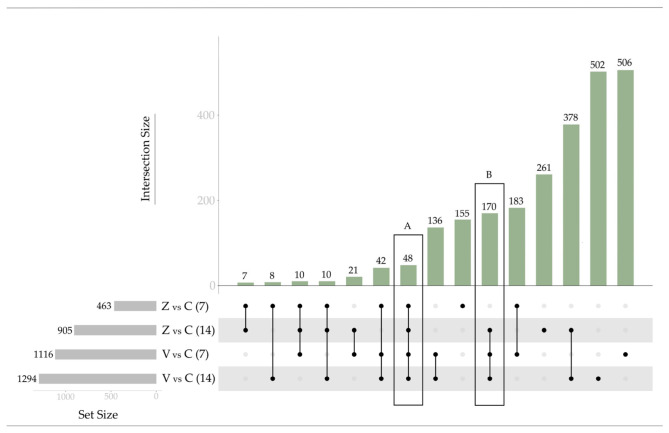
The UpSet plot of cucumber DEGs. The horizontal bars (set size) show the total number of DEGs in each comparison group. The vertical bars (intersection size) show the number of genes unique or common to several comparison groups. Intersection (**A**) corresponds to genes differentially expressed in all compared groups. Intersection (**B**) represents genes common to the *Forc* V03-2g inoculated groups at 7 and 14 dpi and the *Forl* ZUM2407 inoculated group at 14 dpi. Abbreviations: C—untreated healthy plants; V—plants inoculated with *Forc* V03-2g; Z—plants inoculated with *Forl* ZUM2407; numbers in brackets 7 and 14 indicate days post-inoculation (dpi).

To compare the transcriptional response of cucumber plants to *Forc* V03-2g and *Forl* ZUM2407 infection, RNA-seq analysis was performed at 7 and 14 dpi. Differentially expressed genes (DEGs) were identified using the following criteria: adjusted *p*-value < 0.01, log_2_ fold change (log_2_FC) > |1|, and base mean (expression level in normalized counts) > 50. The distribution of DEGs across comparison groups is shown in an UpSet plot (Figure 2).

The total number of cucumbers DEGs differed between groups and time points inoculated with *Forc* V03-2g and *Forl* ZUM2407 strains. At 7 dpi, *Forl* ZUM2407 induced 463 DEGs, which is less than half the number observed in the other comparisons (905–1294 DEGs). Across both time points, *Forc* V03-2g consistently induced a higher number of DEGs (1116 at 7 dpi and 1294 at 14 dpi) compared to *Forl* ZUM2407 (463 at 7 dpi and 905 at 14 dpi). Strain-specific cucumber response DEGs were more abundant in *Forc* V03-2g, with 506 genes at 7 dpi and 502 genes at 14 dpi. In contrast, *Forl* ZUM2407 exhibited just 155 and 261 unique DEGs at 7 and 14 dpi, respectively.

A set of 48 DEGs was common to all four comparison groups (Figure 2A), representing a shared transcriptional response of cucumber to infection by both strains at both time points. An additional set of 170 DEGs was shared among three conditions, being induced with *Forc* V03-2g at both time points and with *Forl* ZUM2407 only at 14 dpi (Figure 2B), indicating delayed defense activation during *Forl* ZUM2407 infection. To characterize the functional composition of these gene sets (Figure 2A,B), genes with the highest log_2_FC values and annotated functions in plant defense responses were listed in Table 1.

The intersection A included genes encoding pathogenesis-related (PR) proteins, such as chitinases, thaumatin-like proteins, and β-1,3-glucanases. Additional components comprised lipoxygenase involved in jasmonate biosynthesis, receptor-like kinases, WRKY transcription factor 71, cytochrome P450, and proteins associated with cell wall structure and remodeling, including expansins, extensins, and wall-associated kinases. At 7 dpi, the magnitude of induction of these genes was consistently lower in plants inoculated with *Forl* ZUM2407 compared to those inoculated with *Forc* V03-2g (e.g., Chitinase 1—1.37 vs. 5.01; Thaumatin-like protein 1b—4.35 vs. 9.51; Cytochrome P450 CYP82D47—1.79 vs. 4.17, respectively). By 14 dpi, these differences for most genes were reduced (e.g., Chitinase 1—5.02 vs. 6.31; Thaumatin-like protein 1b—7.03 vs. 8.15, respectively).

The intersection B included genes associated with oxidative stress responses, cell wall reinforcement and phenylpropanoid biosynthesis, such as multiple peroxidases, 4-coumarate--CoA ligase 2, berberine bridge enzyme-like 18, cytochrome P450 CYP73A100 and dirigent proteins. Additional defense-related genes included chitinase, cytochrome P450 CYP749A22 and WRKY transcription factors (33 and 72A). In plants inoculated with *Forc* V03-2g, these genes exhibited strong induction already at 7 dpi. In contrast, the expression of these genes for plants inoculated with *Forl* ZUM2407 remained at the level of the control untreated plants at 7 dpi (e.g., Peroxidase 2-like—3.02–4.50 vs. “-”; transcription factor WRKY 33—2.21 vs. “-”; Cytochrome P450 CYP749A22—4.91 vs. “-”). By 14 dpi, differences in responses to both strains were reduced to minimum, indicating delayed response to *Forl* ZUM2407 (e.g., Peroxidase 2-like—1.87–3.48 vs. 1.46–2.98; transcription factor WRKY 33—2.60 vs. 2.74; Cytochrome P450 CYP749A22—3.91 vs. 4.26). A complete list of DEGs in all intersections is provided in Table S3.

**Figure 3:**
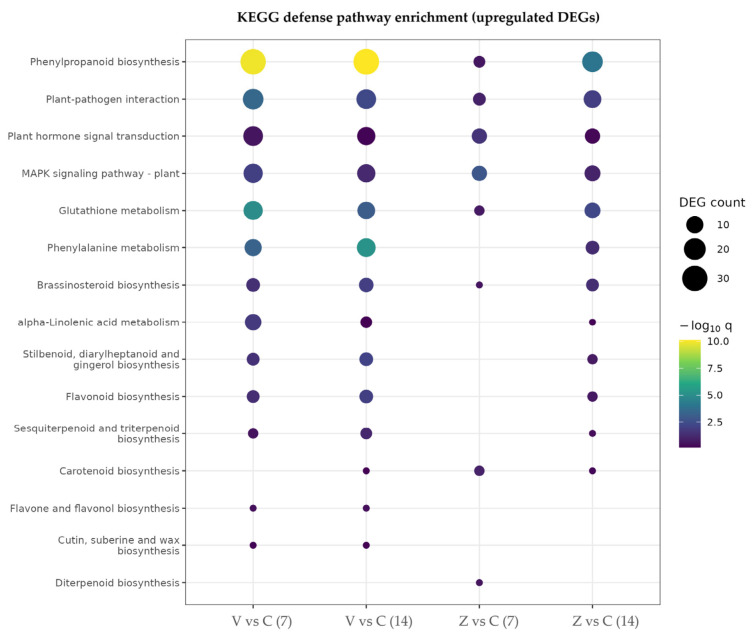
Comparative KEGG defense pathway enrichment analysis in cucumber at 7 and 14 dpi among upregulated DEGs. Dot size denotes the number of DEGs; color denotes -log_10_ (adjusted *p*-value). Abbreviations: C—untreated healthy plants; V—plants inoculated with *Forc* V03-2g; Z—plants inoculated with *Forl* ZUM2407; numbers in brackets 7 and 14 indicate days post-inoculation (dpi).

The pattern represented by intersections A and B is also reflected in cucumber defense pathways across all intersections (Figure 3).

At the pathway level, the cucumber defense response to *Forc* V03-2g was stronger than to *Forl* ZUM2407 at 7 dpi in six pathways: phenylpropanoid biosynthesis, plant–pathogen interaction, plant hormone signal transduction, MAPK signaling, glutathione metabolism, and brassinosteroid biosynthesis. At 7 dpi, during *Forc* V03-2g infection, seven additional defense pathways were induced (phenylalanine metabolism, alpha-linolenic acid metabolism, stilbenoid, flavonoid, sesquiterpenoid, flavone, and cutin biosynthesis), whereas during *Forl* ZUM2407 infection, only two pathways were induced (carotenoid and diterpenoid biosynthesis). Interestingly, phenylpropanoid biosynthesis, which was also highly represented in intersection B (Table 1), was repressed during *Forl* ZUM2407 infection at 7 dpi (Figure S6).

By 14 dpi, the response to *Forl* ZUM2407 had become comparable to that induced by *Forc* V03-2g in terms of DEG number and enriched pathways. Cucumber enriched five of the seven defense pathways (except flavone and cutin biosynthesis) during *Forl* ZUM2407 infection compared to 7 dpi, and phenylpropanoid biosynthesis shifted from repression to induction, reaching a level similar to that in the response to *Forc* V03-2g (Figure 3).

### 3.3. Differential Gene Expression in Tomato Following Inoculation with Forc V03-2g and Forl ZUM2407

**Figure 4:**
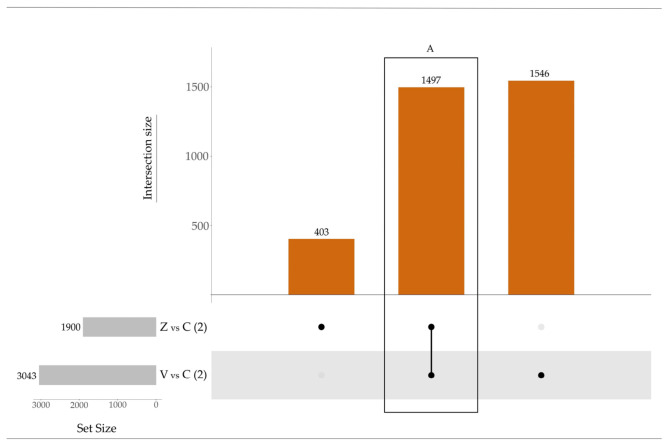
The UpSet plot of tomato DEGs. The horizontal bars (set size) show the total number of DEGs in each comparison group. The vertical bars (intersection size) show the number of genes unique or common to several comparison groups. Intersection (**A**) corresponds to genes differentially expressed in all compared groups. Abbreviations: C—untreated healthy plants; V—plants inoculated with *Forc* V03-2g; Z—plants inoculated with *Forl* ZUM2407; numbers in brackets 2 indicate days post-inoculation (dpi).

To compare the early transcriptional response of tomato plants to *Forc* V03-2g (non-pathogenic on tomato) and *Forl* ZUM2407 (pathogenic on tomato) inoculation, RNA-seq analysis was performed at 2 dpi. DEGs were identified using the same criteria applied for cucumber. The distribution of DEGs across comparison groups is shown in an UpSet plot (Figure 4).

A total of 1900 DEGs were identified in response to *Forl* ZUM2407, whereas *Forc* V03-2g induced 3043 DEGs. Among these, 1497 DEGs were shared between both inoculated groups (Figure 4A). The higher number of DEGs observed in response to the *Forc* V03-2g may indicate a broader activation of host defense pathways, consistent with the resistance of tomato to this strain. To characterize the functional composition of tomato shared response in relation to cucumber response (Table 1), genes with the highest log_2_FC values and annotated functions homologous to genes indicated in cucumber intersection A and B were listed in Table 2.

The intersection A included genes encoding PR genes, chitinases, and β-1,3-glucanases, and genes associated with cell wall remodeling, such as expansins and extensins. In addition, increased expression was observed for peroxidases and WRKY transcription factors (WRKY41, 43, 51, 33 and 75) for both *Forc* V03-2g and *Forl* ZUM2407 strains.

For most genes listed in Table 2, the magnitude of expression was 2-4 times higher (log_2_FC difference 1-2) in response to *Forc* V03-2g than to *Forl* ZUM2407 (e.g., PR-5—4.53 vs. 2.08; glucan endo-1,3-beta-glucosidase—5.42 vs. 3.23; basic endochitinase—4.77 vs. 3.31, respectively). Exceptions to this pattern were extensin and WRKY33 genes, for which expression level were approximately equal in response to *Forc* V03-2g and *Forl* ZUM2407 pretreatment (3.80 vs. 3.89 and 2.36 vs. 2.32, respectively). The same pattern was observed in defense pathways across all intersections, with a higher number of upregulated DEGs in response to *Forc* V03-2g than to *Forl* ZUM2407 (Figures S8 and S9). A complete list of DEGs in all intersections is provided in Supplementary Table S4.

### 3.4. Comparative Transcriptomic Analysis of Cucumber and Tomato Plants Following Inoculation with Forc V03-2g and Forl ZUM2407

Because tomato and cucumber were sampled at different time points (2 dpi vs. 7 and 14 dpi), we compared tomato responses at 2 dpi with cucumber responses at 7 and 14 dpi, focusing on those immune family genes whose number and expression level are increasing or stable during cucumber infection, pointing to increasing or constant modulation of these genes from early to later stages of plant–pathogen interaction. Thus, both the higher number of such genes and their expression levels already in tomato at the early infection stage (2 dpi) compared to cucumber at later stages (7 and 14 dpi) could suggest differences between hosts in response to both pathogens.

Notably, homologs of genes that exhibited delayed upregulation by 14 dpi during *Forl* ZUM2407 infection in cucumber (genes involved in phenylpropanoid biosynthesis, peroxidases and transcription factor WRKY33) were already induced in tomato at 2 dpi after inoculation with *Forl* ZUM2407. Also, the magnitude of chitinase induction in tomato in response to *Forl* ZUM2407 (log_2_ FC range 1.45–5.92) exceeded that observed in cucumber at 7 dpi (log_2_ FC range 1.37–2.12). Moreover, repression in phenylpropanoid biosynthesis observed in cucumber at 7 dpi in response to *Forl* ZUM2407 was not detected in tomato at 2 dpi (Figure S8).

The number of upregulated genes related to immune families in response to infection with *Forc* V03-2g and *Forl* ZUM2407 in both plants represented in Table 3.

For all immune families, the number of DEGs was consistently higher in response to *Forc* V03-2g than to *Forl* ZUM2407 across most comparisons. In cucumber, several families (LRR-RLK/PRR, Class III peroxidase, chitinase PR-3) show a clear temporal pattern of lower induction by *Forl* ZUM2407 than by *Forc* V03-2g at 7 dpi (7 vs. 22, 1 vs. 16, 1 vs. 4, respectively) followed by an increase at 14 dpi (11 vs. 23, 11 vs. 17, 3 vs. 3). In contrast, tomato at 2 dpi already exhibits higher DEG number for these families (for *Forc* V03-2g vs. *Forl* ZUM2407: 21 vs. 28 for LRR-RLK/PRR, 27 vs. 28 for Class III peroxidase, 4 vs. 5 for chitinase PR-3, respectively), compared to cucumber at 7 and 14 dpi in response to both strains, especially in response to *Forl* ZUM2407 at 7 dpi (7, 1 and 1 for these families, respectively). Notably, LRR-RLK/PRR and NLR/R family genes are induced in cucumber to a much lesser extent by *Forl* ZUM2407 than by *Forc* V03-2g throughout infection. In tomato, LRR-RLK/PRR genes show less difference between strains, whereas a clear difference is observed for the NLR/R family (11 vs. 30 for *Forl* ZUM2407 vs. *Forc* V03-2g, respectively). In cucumber, the number of NLR/R genes induced by *Forl* ZUM2407 reaches only 1 at 7 dpi and 0 at 14 dpi.

The overall summary across immune family genes shows that in response to *Forc* V03-2g cucumber induces almost twice as many DEGs than to *Forl* ZUM2407 at 7 dpi (99 vs. 39, respectively), by 14 dpi this difference decreases (89 vs. 53), whereas tomato in response to *Forc* V03-2g and *Forl* ZUM2407 induces higher number of immune family genes than cucumber at later infection stages (172 vs. 125, respectively). A heatmap of cucumber and tomato defense gene expression is provided in Figures S10 and S11. Comparative GO biological process defense enrichment and KEGG defense maps for both plants are represented in Figures S12–S14.

### 3.5. Comparative Transcriptomic Analysis of Forc V03-2g and Forl ZUM2407 During Colonization of Cucumber and Tomato Plants

**Figure 5:**
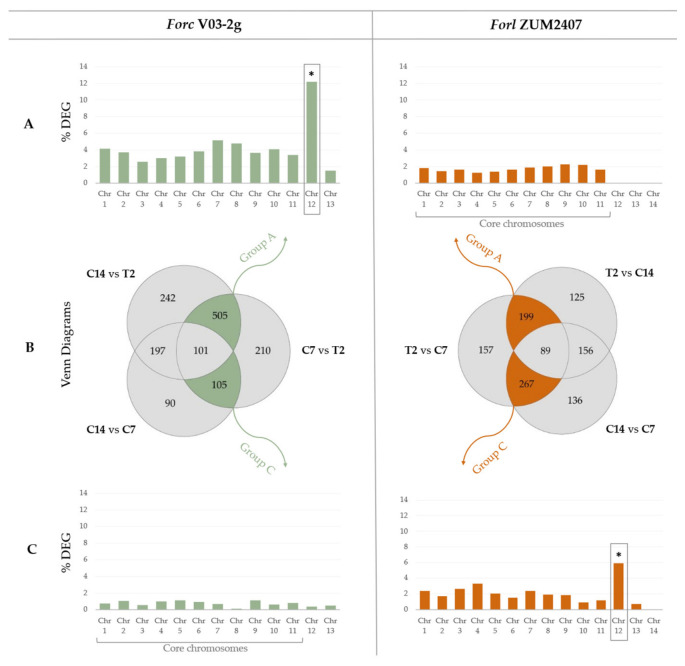
The degree of *Forc* V03-2g and *Forl* ZUM2407 chromosomes involvement in colonization of cucumber and tomato plants. (**A**) Histograms show the percentage of DEGs (highlighted by colored sectors, group A) on a particular chromosome relative to all expressed genes on the same chromosome; (**B**) Venn diagrams show intersecting DEGs between comparison groups; (**C**) histograms show the percentage of DEGs (highlighted by colored sectors, group C) from a particular chromosome relative to all expressed genes on the same chromosome. Black framed bars with an asterisk indicate chromosomes with the highest change in proportion of DEGs depending on the host (Group A) or on dpi in cucumber (Group C). Gray braces indicate 11 *Fusarium oxysporum* core chromosomes. Abbreviations: T2—in tomato, 2 dpi; C7—in cucumber, 7 dpi; C14—in cucumber, 14 dpi; Chr—Chromosomes.

Fungal DEGs associated with host plant or infection stage were identified using three pairwise comparisons performed for each fungal strain in planta: in tomato at 2 dpi vs. in cucumber at 7 dpi (T2 vs. C7), in tomato at 2 dpi vs. in cucumber at 14 dpi (T2 vs. C14), and in cucumber at 14 dpi vs. in cucumber at 7 dpi (C14 vs. C7) (Figure 5).

#### 3.5.1. *Forc* V03-2g and *Forl* ZUM2407 DEGs Distribution Between Core and Accessory Chromosomes

The distribution of fungal DEGs across chromosomes was performed for two intersection groups (Figure 5B) to evaluate the degree of involvement of individual chromosomes in adaptation to changes in living conditions of *Forc* V03-2g and *Forl* ZUM2407. Group A includes genes shared between the C7 vs. T2 and C14 vs. T2 comparisons and reflects the transcriptional response to switching host from tomato to cucumber. The total number of DEGs in this group was 199 for *Forl* ZUM2407, compared to 505 for *Forc* V03-2g. Group C includes genes shared between the T2 vs. C7 and C14 vs. C7 comparisons and reflects the similarity of transcriptional profiles in T2 and C14 as compared to C7. The total number of DEGs in this group was 267 for *Forl* ZUM2407, compared to 105 for *Forc* V03-2g.

The involvement of each chromosome was calculated as the proportion of DEGs assigned to a given group (Figure 5B, A and C groups) on particular chromosome relative to the total number of expressed genes on the same chromosome (Figure 5A,C). The absolute DEG numbers were not used, as larger chromosomes tend to accumulate more DEGs regardless of their specific role in host adaptation.

*Forc* V03-2g showed the highest involvement of accessory chromosome 12 (12.2% DEGs) in the transcriptional response to switching host from tomato to cucumber (Figure 5B, group A), whereas other chromosomes did not exceed 5.2% (Figure 5A). In group C, no chromosome exhibited significant involvement, all accounting for less than 1% (Figure 5C).

*Forl* ZUM2407 in group A showed no involvement of accessory chromosome genes, whereas other chromosomes did not exceed 2.5% DEGs (Figure 5A). In group C, accessory chromosome 12 shows the highest involvement (5.9%), whereas other chromosomes did not exceed 3.5% (Figure 5C).

#### 3.5.2. Transcriptional Profile of *Forc* V03-2g

**Figure 6:**
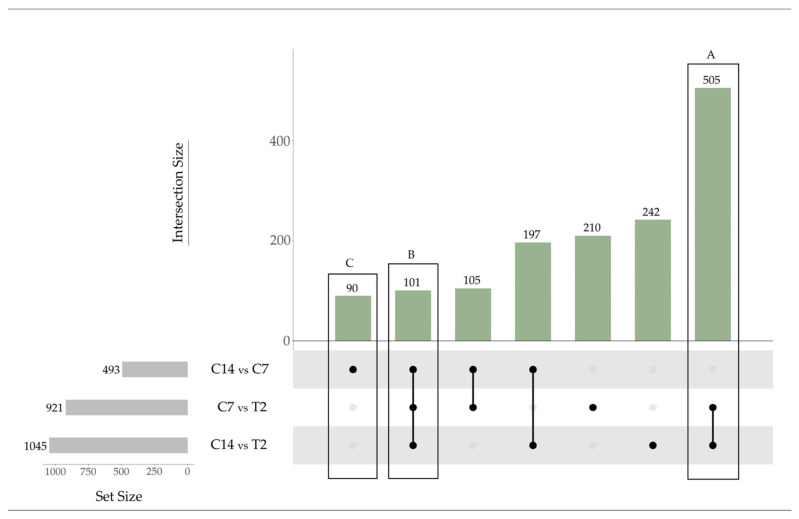
The UpSet plot of *Forc* V03-2g DEGs. The horizontal bars (set size) show the total number of DEGs in each comparison group. The vertical bars (intersection size) show the number of genes unique or common to several comparison groups. Intersection (**A**) corresponds to host-plant-dependent DEGs. Intersection (**B**) corresponds to common DEGs in all compared groups. Intersection (**C**) corresponds to DEGs during symptom development in cucumber. Abbreviations: T2—in tomato, 2 dpi; C7—in cucumber, 7 dpi; C14—in cucumber, 14 dpi.

The number of DEGs in *Forc* V03-2g varied across comparison groups (Figure 6). A total of 493 DEGs were identified in C14 vs. C7, whereas C7 vs. T2 and C14 vs. T2 yielded twice as many DEGs as C14 vs. C7: 921 and 1045, respectively.

Three intersections were selected for analysis (Figure 6). The intersection A, the largest, contained genes differentially expressed between host plants (shared in C7 vs. T2 and C14 vs. T2) and was enriched for accessory chromosome 12 genes (Figure 5A). The intersection B contained DEGs shared across all comparisons (C7 vs. T2, C14 vs. T2, and C14 vs. C7), including genes that are host-plant-dependent but downregulated during cucumber infection, and was also enriched for accessory chromosome 12 genes (Table 4). The intersection C contained DEGs from the C14 vs. C7 comparison and included upregulated genes associated with symptom development during cucumber infection. The intersection C comprised only core chromosome genes (Table 4).

To characterize the functional composition of these intersections, the genes with the highest log_2_FC values that encode secreted proteins were listed in Table 4. For intersections A and B, only genes from accessory chromosomes are represented.

The intersection A included genes encoding carbonic anhydrase, two glucosidase II β-subunit-like proteins, glycosyl hydrolases (families 3 and 31), a necrosis-inducing protein (NPP1), and a protein of unknown function. These genes were consistently upregulated during cucumber infection at 7 and 14 dpi (log_2_FC 2.76–14.00) compared to tomato at 2 dpi, indicating sustained activity. Carbonic anhydrase and the unknown gene showed exceptionally high expression levels (base mean 6423 and 3265, respectively) compared to the housekeeping gene Tef-1α (4668), ranking among the most highly expressed genes in *Forc* V03-2g.

The intersection B included genes encoding SIX6, SIX9, SIX11, SIX13, astacin, Pep1 and six proteins of unknown function. These genes were also upregulated during cucumber infection compared to expression in tomato at 2 dpi, but expression decreased at 14 dpi in cucumber (log_2_FC −1.05 to −3.96). SIX6 and SIX9 showed the lowest expression compared to other genes in intersections A and B (base mean 51 and 64, respectively). An unknown gene ForcV032g.1337 and SIX11 showed the highest expression in intersection (base mean 699 and 804, respectively) and the smallest downregulation in cucumber at 14 dpi (log_2_FC −1.38 and −1.05, respectively).

The intersection C included genes encoding predominantly hydrolytic enzymes, including cellulases (glycosyl hydrolase families 5 and 12), a cellulose-binding acylhydrolase, glycosyl hydrolase family 74, β-glucosidase (SUN family), alkaline phosphatase family proteins, and peptidases (M28 family). These genes showed upregulation at 14 dpi compared to 7 dpi in cucumber (log_2_FC 1.65 to 3.35).

Genes from all intersections not listed in Table 4 are provided in Supplementary Table S5.

#### 3.5.3. Transcriptional Profile of *Forl* ZUM2407

**Figure 7:**
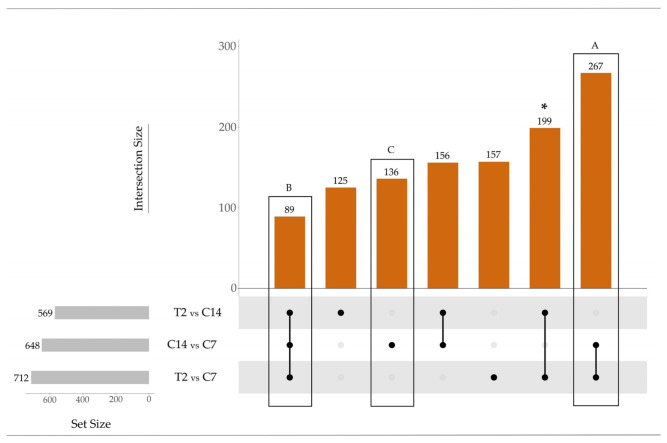
The UpSet plot of *Forl* ZUM2407 DEGs. Horizontal bars (set size) indicate the total number of DEGs in each comparison group. Vertical bars (intersection size) indicate the number of genes unique or common to several comparison groups. Intersection (**A**) corresponds to DEGs coinciding with the delayed defense response in cucumber. Intersection (**B**) corresponds to DEGs common to all comparison groups. Intersection (**C**) corresponds to DEGs associated with symptom development in cucumber. Abbreviations: T2—in tomato, 2 dpi; C7—in cucumber, 7 dpi; C14—in cucumber, 14 dpi. The asterisk indicates the intersection representing DEGs between host plants.

The number of DEGs in *Forl* ZUM2407 varied less across comparison groups (Figure 7) than in *Forc* V03-2g (Figure 6). The T2 vs. C14 comparison yielded the fewest DEGs (569), unlike in *Forc* V03-2g. The T2 vs. C7 and C14 vs. C7 comparisons yielded 712 and 648 DEGs, respectively, with smaller variation than observed in *Forc* V03-2g.

Three intersections were selected for analysis (Figure 7). The intersection A, the largest, contained DEGs coinciding with the delayed defense response in cucumber (shared in T2 vs. C7 and C14 vs. C7) and was enriched for accessory chromosome 12 genes (Figure 5C). The intersection B contained DEGs shared across all comparisons (T2 vs. C7, T2 vs. C14, and C14 vs. C7) but contained only one gene from accessory chromosomes in contrast to *Forc* V03-2g (Table 5). The intersection C contained DEGs from the C14 vs. C7 comparison and included upregulated genes associated with symptom development during cucumber infection. The intersection C comprised only core chromosome genes (Table 5). The intersection representing DEGs between host plants (shared in T2 vs. C7 and T2 vs. C14, indicated with an asterisk) did not contain accessory chromosome genes, unlike in *Forc* V03-2g, and therefore was not represented in the main text but was included in Table S6.

To characterize the functional composition of these intersections, the genes with the highest log_2_FC values that encode secreted proteins or enzymes involved in secondary metabolite biosynthesis were listed in Table 5. For intersections A and B, only genes from accessory chromosomes are represented.

The intersection A included genes encoding an unknown secreted protein, sulfate adenylyltransferase, hydroxymethylglutaryl-CoA reductase, sulfotransferase, geranyl-geranyl and terpene synthases, a tyrosine phosphatase, four cytochrome P450 enzymes, a fungal trichothecene efflux pump, methyltransferase domain and one transcript with no detected open reading frame (ForlZUM2407.1223). These genes were upregulated in tomato at 2 dpi and in cucumber at 14 dpi (log_2_FC 1.69–6.78), compared to their expression in cucumber at 7 dpi, showing similar expression levels at the early stage of tomato infection and the later stage of cucumber infection (absence of log_2_FC in T2 vs. C14). The unknown gene (ForlZUM2407.9438) and one cytochrome P450 (ForlZUM2407.1224) were among the top 100 most highly expressed genes in *Forl* ZUM2407 at both tomato at 2 dpi and cucumber at 14 dpi, and were the most highly expressed among accessory chromosome genes (base mean 682 and 928, respectively, compared to the housekeeping gene Tef-1α (3319)).

**Figure 8:**
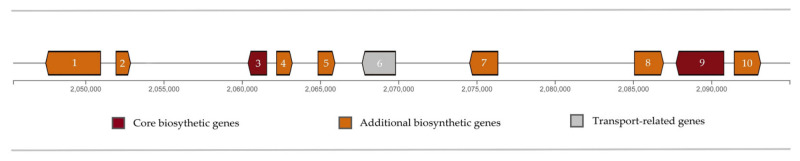
Predicted BGC structure on chromosome 12 of *Forl* ZUM2407. Numbers indicate genes listed in Table 5.

Genes indicated by numbers in brackets 1-10 (Table 5) were predicted to be part of a putative biosynthetic gene cluster (BGC) located on chromosome 12 (Figure 8). This putative BGC was predicted to be involved in terpene biosynthesis and shows low similarity to a characterized BGC in the antiSMASH database (BGC0000688: copalyl diphosphate from *Diaporthe amygdali*; GenBank accession number: AB254160.1).

Notably, this BGC is present in only two sequenced *Ascomycota* genomes, whereas the unknown gene (ForlZUM2407.9438) is found in only three, as shown in Table 6.

The intersection B included the only gene from accessory chromosomes encoding carbonic anhydrase. This gene was downregulated in all compared groups (log_2_FC −1.33 to −2.90) showing the highest expression in cucumber at 7 dpi. A similar expression pattern was observed for its homologous carbonic anhydrase gene in *Forc* V03-2g, but the expression level in *For*l ZUM2407 was significantly lower than in *Forc* V03-2g (base mean 130) compared to the housekeeping gene Tef-1α (3319).

The intersection C included genes encoding predominantly hydrolytic enzymes, including glycosyl hydrolase family 7 (cellulase C), peptidases (S8 family), β-glucosidase (SUN family), glycosyl hydrolase family 10, and a cellulose-binding acylhydrolase. These genes showed upregulation at 14 dpi compared to 7 dpi in cucumber (log2FC 1.23 to 4.40). The number of genes encoding hydrolytic enzymes and their expression level in this intersection were lower than those observed for *Forc* V03-2g under comparable conditions.

Genes from all intersections not listed in Table 5 are provided in Supplementary Table S6.

#### 3.5.4. Validation of Accessory Chromosome Gene Expression Trends by RT-qPCR

**Figure 9:**
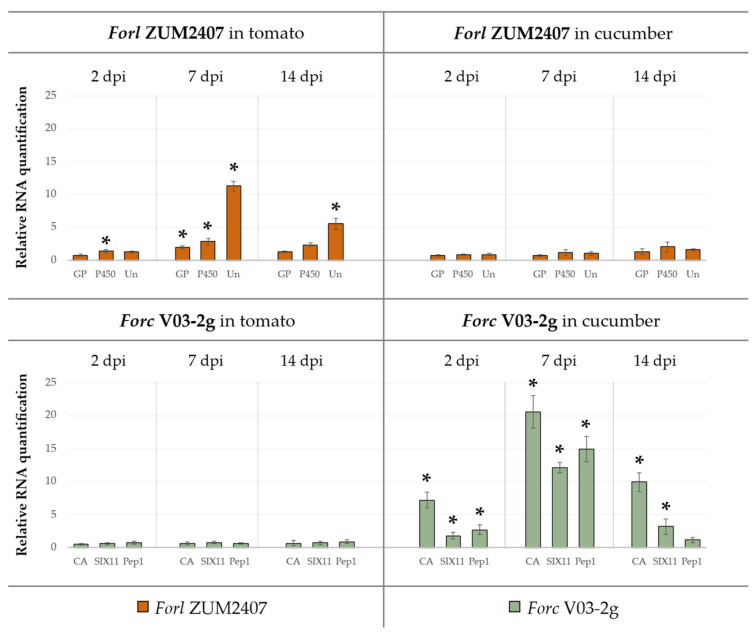
Relative mRNA quantification of highly expressed DEGs on accessory chromosomes of *Forl* ZUM2407 and *Forc* V03-2g. “GP”—belongs to the FPP/GGPP synthase family (ForlZUM2407.1219); “P450”—cytochrome P450 (ForlZUM2407.1224); “Un”—unknown gene (ForlZUM2407.9438); “CA”—carbonic anhydrase (ForcV032g.1401); SIX11—ForcV032g.1368; Pep1—ForcV032g.1322. Asterisks indicate statistically significant differences in gene expression between the two host plants (ANOVA, *p* < 0.05).

The most highly expressed fungal DEGs located on accessory chromosomes were selected to confirm the RNA-seq results and to study their expression at additional time points post-inoculation in tomato (7 and 14 dpi) and cucumber (2 dpi) using RT-qPCR (Figure 9).

For *Forc* V03-2g, the three tested genes (carbonic anhydrase, SIX11, Pep1) were upregulated during cucumber infection, peaking at 7 dpi (RQ: 20.6, 12.1, 14.9, respectively), and declined by 14 dpi. In tomato, expression of these genes remained at background levels (RQ < 1.0) at all time points.

For *Forl* ZUM2407, the three tested genes (FPP/GGPP synthase, cytochrome P450, and the unknown gene (ForlZUM2407.9438)) were upregulated during tomato infection, also peaking at 7 dpi (RQ: 2.8, 1.9, 11.3, respectively). In cucumber, the expression of those genes was low, especially for the unknown gene (ForlZUM2407.9438), which reached values comparable to those in tomato at 2 dpi (RQ: 1.3) only by 14 dpi (RQ: 1.6).

#### 3.5.5. Comparative Analysis of Core Secretome Gene Expression Between *Forc* V03-2g and *Forl* ZUM2407

**Figure 10:**
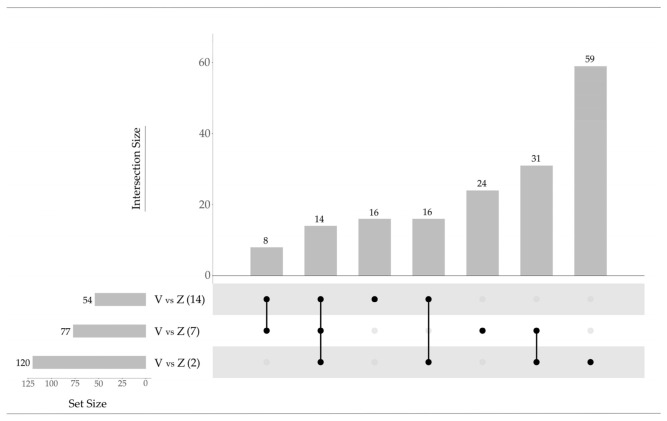
The UpSet plot of *Forc* V03-2g and *Forl* ZUM2407 DEGs encoding secreted proteins. The horizontal bars (set size) show the total number of DEGs in each comparison group. The vertical bars (intersection size) show the number of genes unique or common to several comparison groups. Abbreviations: V—*Forc* V03-2g; Z—*Forl* ZUM2407; (2)—in tomato, 2 dpi; (7)—in cucumber, 7 dpi; (14)—in cucumber, 14 dpi.

To compare core genes expression between *Forc* V03-2g and *Forl* ZUM2407 during infection of tomato and cucumber, RNA-seq reads from both strains were aligned to the eleven core chromosomes of *Forc* V03-2g. Only DEGs encoding predicted secreted proteins were analyzed. The number of DEGs between the two strains varied across comparison groups (Figure 10).

A total of 120 DEGs were identified in tomato at 2 dpi, whereas in cucumber, 77 and 51 DEGs were identified at 7 and 14 dpi, respectively. To characterize the functional composition of these comparison groups, the genes that encode hydrolytic enzymes and other virulence-associated genes were listed in Table 7.

Six pectate lyase genes were differentially expressed across time points. In tomato at 2 dpi, five DEGs were upregulated in *Forl* ZUM2407 (log_2_FC −1.27 to −2.36). In cucumber at 7 dpi, three genes were upregulated in *Forc* V03-2g (log_2_FC 1.41 to 3.88), while the other three showed no differential expression. In cucumber at 14 dpi, two different genes were upregulated in *Forc* V03-2g (log_2_FC 2.80 to 2.87), while the others showed no differential expression.

Eight peptidase genes were differentially expressed. In tomato at 2 dpi, six DEGs were upregulated in *Forl* ZUM2407 (log_2_FC −1.44 to −7.49). In cucumber at 7 dpi, six other DEGs were also upregulated in *Forl* ZUM2407 (log_2_FC −1.89 to −6.49), while one gene was upregulated in *Forc* V03-2g (log_2_FC 2.05). At 14 dpi, the only gene was upregulated in *Forl* ZUM2407 (log_2_FC −1.95), while the others showed no differential expression.

Eight glycosyl hydrolase genes were differentially expressed. In tomato at 2 dpi, seven genes were upregulated in *Forl* ZUM2407 (log_2_FC −1.15 to −9.03). In cucumber at 7 dpi, two genes were upregulated in *Forc* V03-2g (log_2_FC 1.30 to 1.99) and one in *Forl* ZUM2407 (log_2_FC −4.63). In cucumber at 14 dpi, one gene was upregulated in *Forc* V03-2g (log_2_FC 2.02 to 3.12), while the others showed no differential expression.

Ten other virulence-associated genes were differentially expressed. Cupin (log_2_FC −1.19 to −2.58), guanyl-specific ribonuclease F1 (log_2_FC −1.75 to −5.04) and pyridoxamine 5′-phosphate oxidase (log_2_FC –5.53 to −10.02) genes were consistently upregulated in *Forl* ZUM2407 across all time points. Four CFEM domain-encoding genes were upregulated by both strains depending on host and time point, with no differences between strains in cucumber at 14 dpi. Necrosis-inducing protein NPP1 gene was upregulated in *Forl* ZUM2407 in tomato at 2 dpi (log_2_FC −1.61) but showed no differential expression in cucumber. Eukaryotic-type carbonic anhydrase and catalase and peroxidase activity genes were upregulated in *Forc* V03-2g (log_2_FC 2.25 and 2.39, respectively) in cucumber at 7 dpi but were expressed at similar levels at other time points.

Genes from all intersections not listed in Table 7 are provided in Supplementary Table S7.

### 3.6. Integrated Analysis of Plant and Fungal Gene Expression During Cucumber Infection

**Figure 11:**
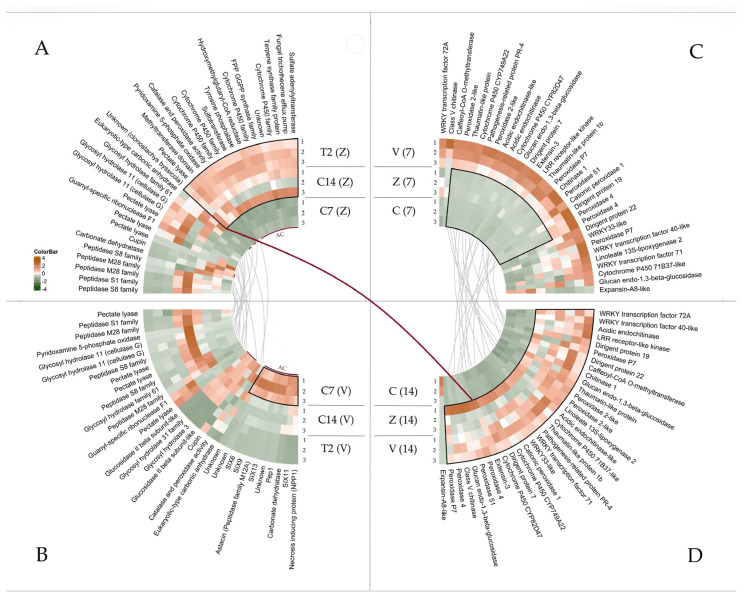
Z-score circos heatmap integrating fungal and cucumber transcriptomic responses during infection. Sectors (**A**,**B**) show fungal responses across host and time points. Sectors (**C**,**D**) show cucumber responses at 7 and 14 dpi, respectively. Numbers 1–3 indicate technical replicates. Black-framed gene groups: sector (**A**)—*Forl* ZUM2407genes highly expressed relative to 7 dpi in cucumber; sector (**B**)—*Forc* V03-2g genes highly expressed at 7 dpi in cucumber; (**C**)—genes representing cucumber response to *Forl* ZUM2407 clustered with control untreated group at 7 dpi; (**D**)—genes representing cucumber response to *Forl* ZUM2407 clustered with response to *Forc* V03-2g at 14 dpi. Red lines highlight accessory chromosome (AC) genes; thin gray lines connect fungal core genome genes/cucumber genes. Thick red line suggests interdependence between groups in (**A**,**D**) sectors. Z-scores were calculated using DESeq2 normalized counts. Brown color represents higher expression levels, green represents lower expression levels (ColorBar). Abbreviations: V—*Forc* V03-2g; Z—*Forl* ZUM2407; C—control untreated cucumber group; T2—in tomato, 2 dpi; C7—in cucumber, 7 dpi; C14—in cucumber, 14 dpi.

The integrated representation of fungal and cucumber transcriptomic responses during the infection process is illustrated as a Z-score circos heatmap in Figure 11.

At 7 dpi, cucumber shows a weak defense response against *Forl* ZUM2407 compared to *Forc* V03-2g (Figure 11C). In *Forc* V03-2g, accessory chromosome 12 genes (SIX effectors, Pep1, carbonic anhydrase, and others) are highly expressed at this time (Figure 11B). In contrast, *Forl* ZUM2407 does not actively express accessory genome genes at 7 dpi. Furthermore, core gene expression differs between the strains: peptidases, cupin, ribonuclease, and others are more active in *Forl* ZUM2407 than in *Forc* V03-2g at 7 dpi.

By 14 dpi, the cucumber response to *Forl* ZUM2407 becomes more similar to that induced by *Forc* V03-2g (Figure 11D). This coincides with *Forl* ZUM2407 upregulating accessory chromosome genes (the predicted BGC and the unknown secreted protein) (Figure 11A). Notably, *Forl* ZUM2407’s delayed accessory gene activation correlates the intensifying plant response, independent of fungal read percentage (Figure S18a). In contrast, *Forc* V03-2g accessory gene expression declines compared to 7 dpi, even though the plant response continues to increase (Figure S19a). However, the differences in expression of core secreted protein genes between the strains diminish by 14 dpi for peptidases, glycosyl hydrolase and other virulence-associated genes.

In tomato at 2 dpi (Figure 11A,B), *Forl* ZUM2407 expresses the same accessory chromosome genes as in cucumber, with an intensity similar to that in cucumber at 14 dpi, coinciding with tomato’s relatively strong defense response. However, a correlation of accessory gene expression and plant defense response was not observed within the tomato group (Figure S18b). In contrast, *Forc* V03-2g shows low expression of accessory chromosome genes during tomato infection and induces stronger defense response compared to Forl ZUM2407 (Figure S19b).

Together, these data reveal distinct accessory gene repertoires (unique to each strain) and distinct core gene transcriptional strategies of both strains during cucumber infection.

## 4. Discussion

In the present study, we analyzed transcriptomic data of cucumber and tomato plants infected with *Forc* V03-2g and *Forl* ZUM2407. Both strains are pathogenic on cucumber, but they differ in aggressiveness on this host. Fungal read proportions during cucumber colonization were similar at 7 dpi (≈1%) but diverged by 14 dpi: 19.0% for *Forc* V03-2g versus 8.0% for *Forl* ZUM2407 (Figure 1B), consistent with the disease index, which was higher for *Forc* V03-2g at both time points. In the present experiment, the disease index values were lower (46.9% and 19.5% at 14 dpi, Figure 1A) than in our previous work [12], where the indexes reached 95% and 62% at 14 dpi, respectively. Nevertheless, the trend of higher aggressiveness of *Forc* V03-2g compared to *Forl* ZUM2407 was the same. Also, the high standard deviation in disease index for Forl ZUM2407 at 14 dpi (19.5 ± 11.1%) can be attributed to Forl ZUM2407 causing less uniform symptom onset on cucumber, in contrast to Forc V03-2g (Figure S1). In tomato at 2 dpi, *Forl* ZUM2407 accounted for 5.3% of reads, whereas *Forc* V03-2g reached only 1.0%, in accordance with its inability to successfully colonize tomato [12,13]. These findings are consistent with other *Fusarium oxysporum* pathosystems where colonization rate correlates with disease progression and outcome [27,28].

In cucumber, both strains induced canonical defense response, but the response was weaker for *Forl* ZUM2407. At 7 dpi, *Forl* ZUM2407 induced only 463 DEGs compared to 1116 for *Forc* V03-2g. By 14 dpi, the numbers of DEGs were 905 and 1294, respectively (Figure 2). Induction levels of defense genes at 7 dpi were consistently lower during *Forl* ZUM2407 infection compared to *Forc* V03-2g. By 14 dpi, the differences diminished. In total, *Forc* V03-2g induced a broader set of immune-related genes and a wider range of defense pathways at 7 dpi than *Forl* ZUM2407 (Figure 3, Table 3), with the difference narrowing by 14 dpi, pointing to a delayed defense response to *Forl* ZUM2407 in cucumber. In many pathosystems, a rapid, strong, and coordinated transcriptional response correlates with resistance, whereas a weaker or delayed response is associated with susceptibility [29,30,31,32,33,34,35]. However, cucumber remains susceptible to both strains regardless of the differences in response strength. This suggests that the activation of defense genes may be manipulated or bypassed by fungal virulence genes, which in this case may determine the outcome of the interaction [36,37,38].

The mechanisms of perception and early signaling pathways were activated in response to both pathogens at 7 dpi in cucumber (Figure S13). Thus, cucumber apparently recognizes *Forl* ZUM2407 as early as *Forc* V03-2g. However, the two pathogens differed at the level of the defense pathway, in particular, in the phenylpropanoid biosynthesis, which was induced by *Forc* V03-2g at 7 dpi, but was repressed by *Forl* ZUM2407 at the same time point and induced only by 14 dpi (Figure 3 and Figure S6). Hence, the cucumber defense delay to *Forl* ZUM2407 is located at the output layer rather than at the perception level. This may suggest that *Forl* ZUM2407 either induces this response more slowly during early colonization (as the less aggressive pathogen on cucumber) or actively suppresses it [39,40].

In tomato at 2 dpi, *Forl* ZUM2407 induced 1900 DEGs, whereas *Forc* V03-2g induced 3043 DEGs, with 1497 shared (Figure 4). Induction levels of defense genes were uniformly lower for *Forl* ZUM2407 compared to *Forc* V03-2g, reflecting the weaker pattern also observed in cucumber. In contrast, *Forc* V03-2g cannot infect tomato and triggers a stronger defense response compared to that observed in cucumber at 7 and 14 dpi across upregulated immune family genes (172 vs. 99 and 89, respectively), particularly for NLR/R (30 vs. 6 and 5, respectively). However, the magnitude of upregulation of chitinases (log_2_FC range 2.49–6.92 in tomato vs. 3.09–6.31 in cucumber) was similar between the two hosts in response to *Forc* V03-2g.

The upregulation of immune family genes, whose number and expression level increase or remain stable from early to later stages of plant–pathogen interaction, was higher for *Forl* ZUM2407 in tomato at 2 dpi than in cucumber at 7 and 14 dpi, in terms of the magnitude of homologous gene induction (e.g., chitinases log_2_FC range 1.45–5.92 at 2 dpi in tomato vs. 1.37–2.12 at 7 dpi, but 1.97–5.02 at 14 dpi in cucumber), in the number of upregulated immune family genes (125 vs. 39 and 52, respectively) and in the phenylpropanoid biosynthesis pathway. These observations may indicate distinct host-dependent patterns of *Forl* ZUM2407 interaction [41].

The differential plant responses observed in cucumber and tomato plants allowed us to compare the transcriptomic strategies of both strains, revealing distinct patterns of core and accessory genome usage. The involvement of accessory chromosome genes in pathogenesis has been confirmed for many formae speciales, including *Fusarium oxysporum (Fo)* f. sp. *lycopersici*, *Fo* f. sp. *melonis*, and *Forc* [7,8,42]. In *Forl* ZUM2407, these genes were expressed at similar levels at 2 dpi in tomato and at 14 dpi in cucumber, and were upregulated compared to their expression in cucumber at 7 dpi (Table 5). Furthermore, qRT-PCR results point to more intense expression in tomato plants at later stages (7 and 14 dpi) than in cucumber (Figure 9). This observation suggests a potential role for *Forl* ZUM2407 accessory chromosomes in pathogenesis on both crops.

Accessory chromosomes of *Forl* ZUM2407 are characterized by the absence of SIX genes and a low content of MIMPs and secreted proteins, which distinguishes *Forl* ZUM2407 from *Forc* V03-2g and other *Fo* wilt pathogens [11,12,43]. Nevertheless, we detected dozens of upregulated genes on chromosome 12 (Figure 5C), where a biosynthetic gene cluster (BGC) predicted to be involved in terpene synthesis was identified. The products of such clusters are often secondary metabolite effectors capable of suppressing immunity or modifying host plant physiology [36,44,45,46]. Interestingly, this BGC is present in only two sequenced *Forl* strains (ZUM2407 and CL57) and in one endophytic *Fo* strain, Fo59, which reportedly can cause mild rot symptoms on tomato and is closely related to *Forl* CL57 [47]. This suggests that the BGC may be characteristic of *Fo* tomato root rot pathogens.

We also identified a gene encoding a secreted protein localized on the accessory, the repeat-rich flank of chromosome 6 [12]. This gene was expressed at a level (base mean 682 vs. 3319 for Tef1α) comparable to *Forc* V03-2g effector genes (e.g., SIX11 with base mean 804 vs. 4668 for Tef1α) (Table 4 and Table 5). Moreover, its expression pattern in tomato (RQ 1.3, 11.3, 5.5 at 2, 7, and 14 dpi, respectively) was similar to that of *Forc* V03-2g effectors in cucumber (e.g., for SIX11: RQ 1.8, 12.1, 3.2, respectively), pointing to similar time-dependent roles in pathogenesis on their original hosts. Interestingly, this gene was not found in genomes that contained the BGC but was detected in three endophytic *Clonostachys* strains with 91% nucleotide identity (Table 6), raising the possibility of horizontal gene transfer between *Clonostachys* and *Fusarium* [48]. Nevertheless, convergent evolution or ancestral polymorphism cannot be excluded without broader phylogenetic analysis based on more complete genomic databases.

However, the actively used secretome content in *Forl* ZUM2407 accessory chromosomes is significantly lower than in *Forc* V03-2g (2 vs. 19) (Table 4 and Table 5). This difference, observed during cucumber infection, may emphasize *Forc* V03-2g’s greater adaptability to its original host compared to *Forl* ZUM2407. Nevertheless, a lower content of *Forl* ZUM2407’s potential effector genes may reduce the likelihood of being recognized by the NLR receptors of a particular host [49,50]. Whether this contributes to the strain’s host range remains unclear, as host recognition also involves PRR receptors and other factors. Notably, expression of the same genes in both host plants may indicate their broader host compatibility, potentially enabling *Forl* ZUM2407 to use them during interaction with both hosts [51,52].

The dynamics of potential virulence gene expression in both strains are consistent with data indicating that their activation is determined by the environment in which the fungus resides [53]. This is observed in *Forc* V03-2g during tomato infection, where its virulence genes show low expression. Similar data have been reported for *Fo* f. sp. *cubense*, where effector expression was suppressed in an incompatible interaction [54]. Likewise, *Forl* ZUM2407 exhibits delayed expression of its potential virulence genes during cucumber infection and show higher expression level of these genes in its original host.

Despite the emphasis on accessory chromosome genes, the fact that *Forl* ZUM2407 colonizes cucumber at 7 dpi at a similar level to *Forc* V03-2g (1%) without actively using its accessory genome points to the predominant role of core genes in the infection process. The expression differences observed between strains point to distinct core gene expression patterns in the same environment (Table 7). Moreover, the importance of core genes for early root colonization (ERC) has been reported in *Fo*, where ERC genes play a basic role in endophytic colonization and multi-host compatibility [55]. Also, *Forl* ZUM2407’s greater reliance on core genome functions at 7 dpi in cucumber, together with the repression of phenylpropanoid biosynthesis at 7 dpi, may suggest a potential specific role of these genes in immune suppression. Nevertheless, accessory chromosome gene expression may be necessary for expanding the colonization area initially provided by core genes [56], as observed in *Forl* ZUM2407 by 14 dpi in cucumber, where the delayed plant defense response was accompanied by a delayed expression of potential virulence genes. However, the delayed or interrupted expression of such genes, and their compatibility with the host, may be one of the reasons for an incompatible reaction for *Forc* V03-2g on tomato [10].

Nevertheless, the functional significance of the described specific to *Forl* ZUM2407 and *Forc* V03-2g accessory genes in pathogenesis should be confirmed in further studies.

## 5. Conclusions

*Forc* V03-2g and *Forl* ZUM2407 employ distinct accessory gene repertoires (unique to each strain) and distinct core gene transcriptional strategies during cucumber infection. Cucumber plants recognize both pathogens but show a delayed reaction to *Forl* ZUM2407. *Forc* V03-2g relies on early, strong activation of accessory chromosome-encoded SIX and other potential secreted effectors. In contrast, *Forl* ZUM2407 initially depends on core genes, with delayed activation of accessory chromosome genes that encode potential effectors distinct from those in *Forc* V03-2g. The potential effectors of *Forl* ZUM2407 are also observed during tomato infection, suggesting they function on both hosts, with differences in timing and intensity of expression. Nevertheless, future functional studies, including gene knockouts and complementation assays, are needed to validate the role of the identified genes in host specificity and broad host infection.

## Figures and Tables

**Table 1:** Cucumber plant DEGs representing defense response to *Forc* V03-2g and *Forl* ZUM2407 strains.

Gene ID	Annotation	Base Mean	log_2_FC			
			V vs. C (7)	Z vs. C (7)	V vs. C (14)	Z vs. C (14)
Reference housekeeping gene						
XM_011659465.2	Actin-7	23,681	-	-	-	-
Intersection A						
XM_031887505.1	Chitinase 1	13,147	5.01	1.37	6.31	5.02
XM_004145868.3	Acidic endochitinase-like	2639	4.94	1.95	3.77	2.89
XM_031885050.1	Acidic endochitinase	5740	5.18	2.12	3.09	1.97
XM_004139191.2	Pathogenesis-related protein PR-4	38,602	6.73	3.09	4.96	4.79
XM_004137802.3	Thaumatin-like protein 1b	15,969	9.51	4.35	8.15	7.03
XM_004143878.3	Thaumatin-like protein	8845	7.42	2.76	4.17	3.46
XM_031881428.1	Glucan endo-1,3-beta-glucosidase	2350	9.81	4.40	5.92	6.53
XM_004142087.3	Linoleate 13S-lipoxygenase 2-1	2285	1.61	1.17	1.60	1.36
XM_004146816.3	LRR receptor-like kinase	398	3.27	1.37	2.56	1.60
NM_001305739.1	WRKY transcription factor 71	200	2.26	1.45	2.44	2.38
XM_004147755.3	Cytochrome P450 CYP82D47	2546	4.17	1.79	3.03	2.52
NM_001280621.1	Expansin-A8-like	527	−1.32	−1.03	−6.48	−6.63
XM_004139697.3	Extensin-3	46,861	5.71	3.22	3.02	3.22
XM_004145562.3	Wall-associated receptor kinase 16	531	1.86	1.58	2.66	2.49
Intersection B						
NM_001305682.1 *	Peroxidase 2-like	19,404	4.50	-	1.87	1.46
XM_004143685.3 *	Peroxidase 2-like	2087	3.02	-	3.48	2.98
XM_004138748.3 *	Peroxidase 51	122	6.60	-	3.11	3.66
XM_031886944.1	Peroxidase 4	56	3.89	-	10.13	10.17
XM_004149318.3	Peroxidase 4	289	3.89	-	5.86	6.29
XM_004150558.3	Peroxidase P7	1655	2.77	-	3.43	2.33
XM_004145255.3 *	Cationic peroxidase 1	616	5.55	-	2.48	2.78
XM_004135847.3 *	4-coumarate--CoA ligase 2	400	2.65	-	4.17	4.24
XM_004135674.3 *	Berberine bridge enzyme-like 18	3810	4.44	-	5.01	4.64
XM_004142109.3	Dirigent protein 7	747	2.64	-	3.69	3.09
XM_011655262.2	Dirigent protein 22	1680	1.52	-	2.39	2.16
XM_004144544.2	Dirigent protein 19	118	1.69	-	2.59	2.63
XM_004146651.3	Class V chitinase	190	2.22	-	3.84	4.23
XM_004135381.3	Cytochrome P450 CYP749A22	367	4.91	-	3.91	4.26
XM_011650668.2 *	Cytochrome P450 CYP73A100	335	3.59	-	4.71	5.28
XM_031886484.1	WRKY transcription factor 33	96	2.21	-	2.60	2.74
XM_004152487.3	WRKY transcription factor 72A	356	1.26	-	1.80	1.99

**Note**: C—untreated healthy plants; V—plants inoculated with *Forc* V03-2g; Z—plants inoculated with *Forl* ZUM2407; numbers in brackets 7 and 14 indicate days post-inoculation (dpi). Positive/negative Log2FC indicate upregulated/downregulated genes in inoculated groups (V, Z) relative to control (C). “-” indicates genes showing no changes in expression level relative to C. “Base mean”—expression level in normalized counts. Reference housekeeping gene serves as a guideline for the magnitude of DEGs expression. Asterisks indicate genes involved in phenylpropanoid biosynthesis. Relative expression of represented genes normalized to housekeeping genes and their correlation with fungal read percentage are shown in Figures S5a and S5b, respectively.

**Table 2:** Tomato plant DEGs representing defense response to *Forc* V03-2g and *Forl* ZUM2407 strains.

Gene ID	Annotation	Base Mean	log_2_FC	
			V vs. C (2)	Z vs. C (2)
Reference housekeeping gene				
NM_001308447.1	actin-7	32,007	-	-
Intersection A				
XM_004240042.5	Acidic endochitinase	452	2.49	1.45
XM_004248589.4	Basic endochitinase	41,994	4.77	3.31
NM_001279329.2	Chitinase	294	6.92	5.92
NM_001247475.2	Chitinase	3766	6.35	5.18
NM_001330783.1	Pathogenesis-related protein PR-5	39,202	4.53	2.08
NM_001323319.1	Pathogenesis-related protein STH-2	32,456	7.62	6.07
XM_004252973.5	Pathogenesis-related protein STH-2	1342	5.37	4.79
NM_001247193.2	Pathogenesis-related protein STH-2	10,589	7.57	6.13
XM_004237697.5	Pathogen-related protein	8497	4.11	2.87
XM_069287956.1	Expansin-like B1	355	9.30	8.99
XM_004245775.5	Expansin-like B1	1799	5.72	3.44
XM_004245774.5	Expansin-like B1	1803	6.66	4.97
XM_010322197.2	Extensin	70,686	3.80	3.89
XM_004237723.5	Extensin-3-like	69,148	4.29	3.18
NM_001247876.2	Glucan endo-1,3-beta-glucosidase B	705	5.42	3.23
XM_004249007.5 *	Cationic peroxidase 1	27,026	2.94	2.58
XM_004240007.5 *	Cationic peroxidase 1-like	342	6.99	5.17
XM_069298155.1 *	Cationic peroxidase 1-like	371	7.42	5.22
XM_069290738.1 *	Lignin-forming anionic peroxidase	155	3.53	2.69
XM_004250354.5 *	Lignin-forming anionic peroxidase	1630	5.54	4.14
XM_069298142.1	WRKY transcription factor 41	96	3.29	2.29
XM_004233537.5	WRKY transcription factor 43	836	8.04	7.18
XM_004237793.4	WRKY transcription factor 51	364	4.31	2.27
XM_004246260.5	WRKY transcription factor 33	2351	2.36	2.32
NM_001323315.1	WRKY transcription factor 75	2844	4.33	3.41

**Note**: C—untreated healthy plants; V—plants inoculated with *Forc* V03-2g; Z—plants inoculated with *Forl* ZUM2407; numbers in brackets 2 indicate days post-inoculation (dpi). Positive/negative Log_2_FC indicate upregulated/downregulated genes in inoculated groups (V, Z) relative to control (C). “Base mean”—expression level in normalized counts. Reference housekeeping gene serves as a guideline for the magnitude of DEGs expression. Asterisks indicate genes involved in phenylpropanoid biosynthesis. Relative expression of represented genes normalized to housekeeping genes and their correlation with fungal read percentage are shown in Figure S7a,b.

**Table 3:** Number of upregulated DEGs related to immune families in cucumber and tomato plants inoculated with *Forc* V03-2g and *Forl* ZUM2407.

Immune Family	Cucumber				Tomato	
	V vs. C (7)	Z vs. C (7)	V vs. C (14)	Z vs. C (14)	V vs. C (2)	Z vs. C (27)
LRR-RLK/PRR	22	7	23	11	28	21
NLR/R-gene	6	1	5	0	30	11
WRKY TF	13	11	13	9	19	14
Class III peroxidase	16	1	17	11	28	27
Chitinase (GH19, PR-3)	4	1	3	3	5	4
Chitinase-related (GH18/CBM)	4	5	3	3	7	6
beta-1,3-glucanase (PR-2)	5	2	5	3	9	6
Thaumatin (PR-5)	4	3	3	2	5	5
PR-10/Bet v 1	3	0	1	1	8	8
PR-1 (SA marker)	3	3	3	2	1	1
ET pathway (ACS/ERF)	12	4	5	5	24	18
JA pathway (LOX/AOS/JAZ)	6	1	2	1	6	3
SA biosynthesis (ICS/PAL)	1	0	6	2	2	1
Summary across families	99	39	89	53	172	125

**Note**: C—untreated healthy plants; V—plants inoculated with *Forc* V03-2g; Z—plants inoculated with *Forl* ZUM2407; numbers in brackets 2, 7 and 14 indicate days post-inoculation (dpi).

**Table 4:** *Forc* V03-2g DEGs representing time- and host-dependent response.

Gene ID *	Annotation	Chr	Base Mean	SigP	log_2_FC		
					C7 vs. T2	C14 vs. T2	C14 vs. C7
Reference housekeeping gene							
ForcV032g.10137	Translation elongation factor (Tef) 1α	6	4668	-	-	-	-
Intersection A							
ForcV032g.1401	Carbonic anhydrase	12	6423	+	14.00	12.17	-
ForcV032g.1319	Glucosidase II beta subunit-like	12	198	+	9.37	9.21	-
ForcV032g.1332	Glucosidase II beta subunit-like	12	139	+	8.82	8.79	-
ForcV032g.1288	Glycosyl hydrolase 3 family	12	819	+	2.76	2.80	-
ForcV032g.1318	Glycosyl hydrolase 31 family	12	602	+	7.06	7.03	-
ForcV032g.1307	Necrosis-inducing protein (NPP1)	12	404	+	10.68	9.84	-
ForcV032g.1402	Unknown	12	3265	+	13.00	11.27	-
Intersection B							
ForcV032g.1380	Astacin (Peptidase family M12A)	12	72	+	8.62	5.94	−2.68
ForcV032g.1322	Pep1	12	624	+	6.99	3.35	−3.64
ForcV032g.1368	SIX11	12	804	+	8.58	7.53	−1.05
ForcV032g.1321	SIX13	12	253	+	9.58	5.82	−3.75
ForcV032g.1354	SIX6	12	51	+	4.11	1.79	−2.32
ForcV032g.1316	SIX9	12	64	+	6.55	4.01	−2.54
ForcV032g.1337	Unknown	12	699	+	4.87	3.49	−1.38
ForcV032g.1389	Unknown	12	74	+	8.75	5.18	−3.57
ForcV032g.1298	Unknown	12	205	+	10.22	6.77	−3.46
ForcV032g.1310	Unknown	12	283	+	7.47	4.36	−3.11
ForcV032g.1303	Unknown	12	178	+	9.08	5.12	−3.96
ForcV032g.1312	Unknown	12	81	+	8.89	5.26	−3.63
Intersection C							
ForcV032g.5475	Alkaline phosphatase family	3	42	+	-	-	2.51
ForcV032g.6816	Beta-glucosidase (SUN family)	4	128	+	-	-	1.65
ForcV032g.8080	Cellulose-binding lipase acylhydrolase	5	111	+	-	-	2.63
ForcV032g.487	Glycoside hydrolase family 74 protein	10	612	+	-	-	3.31
ForcV032g.4992	Glycosyl hydrolase 12 (cellulase H)	2	102	+	-	-	2.78
ForcV032g.543	Glycosyl hydrolase 5 (cellulase A)	10	375	+	-	-	3.04
ForcV032g.7661	Pectate lyase	4	58	+	-	-	3.35
ForcV032g.10674	Peptidase M28 family	7	382	+	-	-	2.16
ForcV032g.472	Unknown	10	146	+	-	-	2.11

**Note**: T2—in tomato, 2 dpi; C7—in cucumber, 7 dpi; C14—in cucumber, 14 dpi. Positive log_2_FC values indicate higher expression in the first-named group (e.g., C7 in “C7 vs T2”), negative values indicate higher expression in the second-named group. “Chr”—chromosome; “Base mean”—expression level in normalized counts; “SigP”—signal peptide; “+” indicates presence of a signal peptide; “-” indicate absence of a signal peptide or log_2_FC. Reference housekeeping gene serves as a guideline for the magnitude of DEGs expression. * Sequences of these genes are provided in Table S5. Relative expression of represented genes normalized to housekeeping genes and their correlation with fungal read percentage are shown in Figure S15a,b.

**Table 5:** *Forl* ZUM2407 DEGs representing time- and host-dependent response.

Gene ID *	Annotation	Chr	Base Mean	SigP	log_2_FC		
					T2 vs. C7	T2 vs. C14	C14 vs. C7
Reference housekeeping gene							
ForlZUM2407.10198	Translation elongation factor (Tef) 1α	7	3319	-	-	-	-
Intersection A							
ForlZUM2407.9438	Unknown	6	682	+	5.42	-	5.88
ForlZUM2407.1214	Sulfate adenylyltransferase	12	245	-	1.69	-	1.76
ForlZUM2407.1217	Hydroxymethylglutaryl-CoA reductase (1)	12	278	-	2.73	-	2.39
ForlZUM2407.1218	Sulfotransferase (2)	12	296	-	3.36	-	3.05
ForlZUM2407.1219	Belongs to FPP/GGPP synthase family (3)	12	431	-	3.21	-	2.89
ForlZUM2407.1220	Tyrosine phosphatase (4)	12	152	-	3.12	-	2.70
ForlZUM2407.1221	Belongs to the cytochrome P450 family (5)	12	244	-	2.14	-	1.96
ForlZUM2407.1222	Fungal trichothecene efflux pump (6)	12	55	-	3.19	-	3.19
ForlZUM2407.1223	Unknown	12	121	-	1.95	-	2.33
ForlZUM2407.1224	Belongs to the cytochrome P450 family (7)	12	928	-	3.33	-	2.90
ForlZUM2407.1225	Belongs to the cytochrome P450 family (8)	12	145	-	2.78	-	2.64
ForlZUM2407.1226	Terpene synthase family protein (9)	12	258	-	3.84	-	3.88
ForlZUM2407.1227	Belongs to the cytochrome P450 family (10)	12	428	-	2.53	-	2.71
ForlZUM2407.1293	Methyltransferase domain	13	210	-	6.78	-	6.54
Intersection B							
ForlZUM2407.1208	Carbonic anhydrase	12	130	+	−2.90	−1.33	−1.57
Intersection C							
ForlZUM2407.9605	Glycosyl hydrolase 7 (cellulase C) family	7	110	+	-	-	3.28
ForlZUM2407.3251	Peptidase S8 family	1	67	+	-	-	3.19
ForlZUM2407.3136	Peptidase S8 family	1	275	+	-	-	1.23
ForlZUM2407.8460	Beta-glucosidase (SUN family)	5	65	+	-	-	2.05
ForlZUM2407.7110	Glycosyl hydrolase family 10	4	62	+	-	-	4.40
ForlZUM2407.6168	Cellulose-binding lipase acylhydrolase	4	68	+	-	-	3.39

**Note**: T2—in tomato, 2 dpi; C7—in cucumber, 7 dpi; C14—in cucumber, 14 dpi. Positive log_2_FC values indicate higher expression in the first-named group (e.g., T2 in “T2 vs. C7”), negative values indicate higher expression in the second-named group. “Chr”—chromosome; “Base mean”—expression level in normalized counts; “SigP”—signal peptide; “+” indicates presence of a signal peptide; “-” indicates absence of a signal peptide or log_2_FC. Reference housekeeping gene serves as a guideline for the magnitude of DEGs expression. * Sequences of these genes are provided in Table S6. Relative expression of represented genes normalized to housekeeping genes and their correlation with fungal read percentage are shown in Figure S16a,b.

**Table 6:** Nucleotide sequence similarity of the predicted BGC and the unknown gene (ForlZUM2407.9438) among sequenced *Ascomycota* strains.

Strain	Accession Number	Cover	Identity
Predicted biosynthetic gene cluster			
*Forl* ZUM2407	GCA_048165035.1	100	100
*Forl* CL57	GCA_000260155.3	95	95
*Fusarium oxysporum Fo*59	GCA_014324765.1	83	95
Other *Ascomycota* strains	-	<22	<82
Unknown gene (ForlZUM2407.9438)			
*Forl* ZUM2407	GCA_048165035.1	100	100
*Clonostachys byssicola*	GCA_902006505.2	100	91.98
*Clonostachys rosea* HOCR18	GCA_019843565.1	99	91.94
*Clonostachys rosea* HWLR12	GCA_019843725.1	99	91.94
Other *Ascomycota* strains	-	-	-

**Note**: NCBI BLAST search was performed on 25 May 2026.

**Table 7:** Core DEGs encoding secreted proteins in *Forc* V03-2g and *Forl* ZUM2407, with time- and host-dependent differences.

Gene_id *	Annotation	Chr	SigP	Base Mean	log_2_FC		
					V vs. Z (T2)	V vs. Z (C7)	V vs. Z (C14)
Reference housekeeping gene							
Foxcore.10632	Translation elongation factor (Tef) 1α		-	4055	-	-	-
Pectinases							
Foxcore.8485	Pectate lyase	5	+	615	−1.46	1.41	-
Foxcore.13371	Pectate lyase	9	+	64	−1.27	3.88	-
Foxcore.988	Pectate lyase	11	+	76	−2.13	-	2.80
Foxcore.12888	Pectate lyase	8	+	79	−2.36	-	2.87
Foxcore.594	Pectate lyase D	10	+	92	−2.07	-	-
Foxcore.8136	Pectate lyase B precursor	4	+	530	-	3.20	-
Peptidases							
Foxcore.3866	Peptidase M28 family	2	+	374	−2.40	−2.48	-
Foxcore.476	Peptidase S1 family	10	+	1256	−3.42	−2.19	-
Foxcore.2098	Peptidase S8 family	1	+	726	−7.49	−6.49	-
Foxcore.8024	Peptidase S8 family	4	+	140	−1.44	2.05	-
Foxcore.1226	Peptidase M28 family	11	+	222	−2.79	-	-
Foxcore.11210	Peptidase M28 family	7	+	447	-	−1.89	-
Foxcore.13332	Peptidase S8 family	9	+	410	-	−2.05	-
Foxcore.189	Peptidase S8 family	10	+	90	−2.10	−3.10	−1.95
Glycosyl hydrolases							
Foxcore.10917	Glycosyl hydrolase 11 (cellulase G)	6	+	61	−9.03	−4.63	-
Foxcore.8269	Glycosyl hydrolase family 3	4	+	83	−3.43	1.99	-
Foxcore.7572	Glycosyl hydrolase family 61	4	+	864	−2.16	1.30	-
Foxcore.9517	Glycosyl hydrolase family 10	5	+	69	−2.10	-	-
Foxcore.12858	Glycosyl hydrolase family 61	8	+	210	−1.74	-	-
Foxcore.1948	Glycosyl hydrolases family 16	1	+	53	−1.15	-	-
Foxcore.13459	Glycosyl hydrolase 11 (cellulase G)	9	+	87	−2.29	-	-
Foxcore.705	Glycosyl hydrolase family 115	11	+	52	-	-	2.02
Other virulence-associated genes							
Foxcore.513	Cupin	10	+	264	−1.19	−2.58	−1.41
Foxcore.487	Guanyl-specific ribonuclease F1	10	+	58	−1.75	−5.04	−3.20
Foxcore.13734	Pyridoxamine 5′-phosphate oxidase	9	+	146	−10.02	−5.53	−9.11
Foxcore.3977	CFEM domain	2	+	1673	−1.29	−1.29	-
Foxcore.10680	CFEM domain	6	+	787	−2.05	1.42	-
Foxcore.4899	CFEM domain	2	+	333	−3.20	-	-
Foxcore.10482	CFEM domain	6	+	259	-	1.58	-
Foxcore.5088	Necrosis-inducing protein NPP1	2	+	66	−1.61	-	-
Foxcore.12227	Eukaryotic-type carbonic anhydrase	8	+	142	-	2.25	-
Foxcore.764	Catalase and peroxidase activity	11	+	113	-	2.39	-

**Note**: V—*Forc* V03-2g; Z—*Forl* ZUM2407; T2—in tomato, 2 dpi; C7—in cucumber, 7 dpi; C14—in cucumber, 14 dpi. Positive log_2_FC values indicate higher expression in the first-named group (e.g., V in “V vs. Z”), negative values indicate higher expression in the second-named group. “Chr”—chromosome; “Base mean”—expression level in normalized counts; “SigP”—signal peptide; “+” indicates presence of a signal peptide; “-” indicates absence of a signal peptide or log_2_FC. Reference housekeeping gene serves as a guideline for the magnitude of DEGs expression. * Sequences of these genes and alignment results between both strains are provided in Table S7. Relative expression of represented genes normalized to housekeeping genes and their correlation with fungal read percentage are shown in Figure S17a,b.

## Data Availability

The original contributions presented in the study are included in the article and Supplementary Materials; further inquiries can be directed to the corresponding authors.

## Supplementary Materials

The following supporting information can be downloaded at: https://www.mdpi.com/article/10.3390/jof12070540/s1.
