# Supplementary material for: Transcriptomic Differences Between Two Fusarium oxysporum Formae Speciales During Cucumber Infection
Source: J Fungi (Basel). 2026 Jul 22;12(7):540. doi: 10.3390/jof12070540 (PMC13412168; doi:10.3390/jof12070540)
Supplement: Supplementary file 1 [file jof-12-00540-s001.zip › jof-4415985-supplementary figures S1-S19.pdf]

Transcriptomic Differences Between Two *Fusarium oxysporum* Formae Speciales During Cucumber Infection

Ernest Nailevich Komissarov [1,*], Alfred Onele Obinna [1], Inna Alexandrovna Abdeeva [2], Mariya Vladimirovna Mokryakova [2], Sergey Alexandrovich Bruskin [2,3] and Shamil Zavdatovich Validov [1]

[1] Laboratory of Molecular Genetics and Microbiology Methods, Kazan Scientific Center of the Russian Academy of Sciences, 420111 Kazan, Russia; donjay.ao@gmail.com (A.O.O.); sh.validov@knc.ru (S.Z.V.)

[2] Vavilov Institute of General Genetics, Russian Academy of Sciences, 119991 Moscow, Russia; insaz@vigg.ru (I.A.A.); mokryakova@vigg.ru (M.V.M.); brouskin@vigg.ru (S.A.B.)

[3] Moscow Center for Advanced Studies, 123592 Moscow, Russia

* Correspondence: e.komissarov@knc.ru

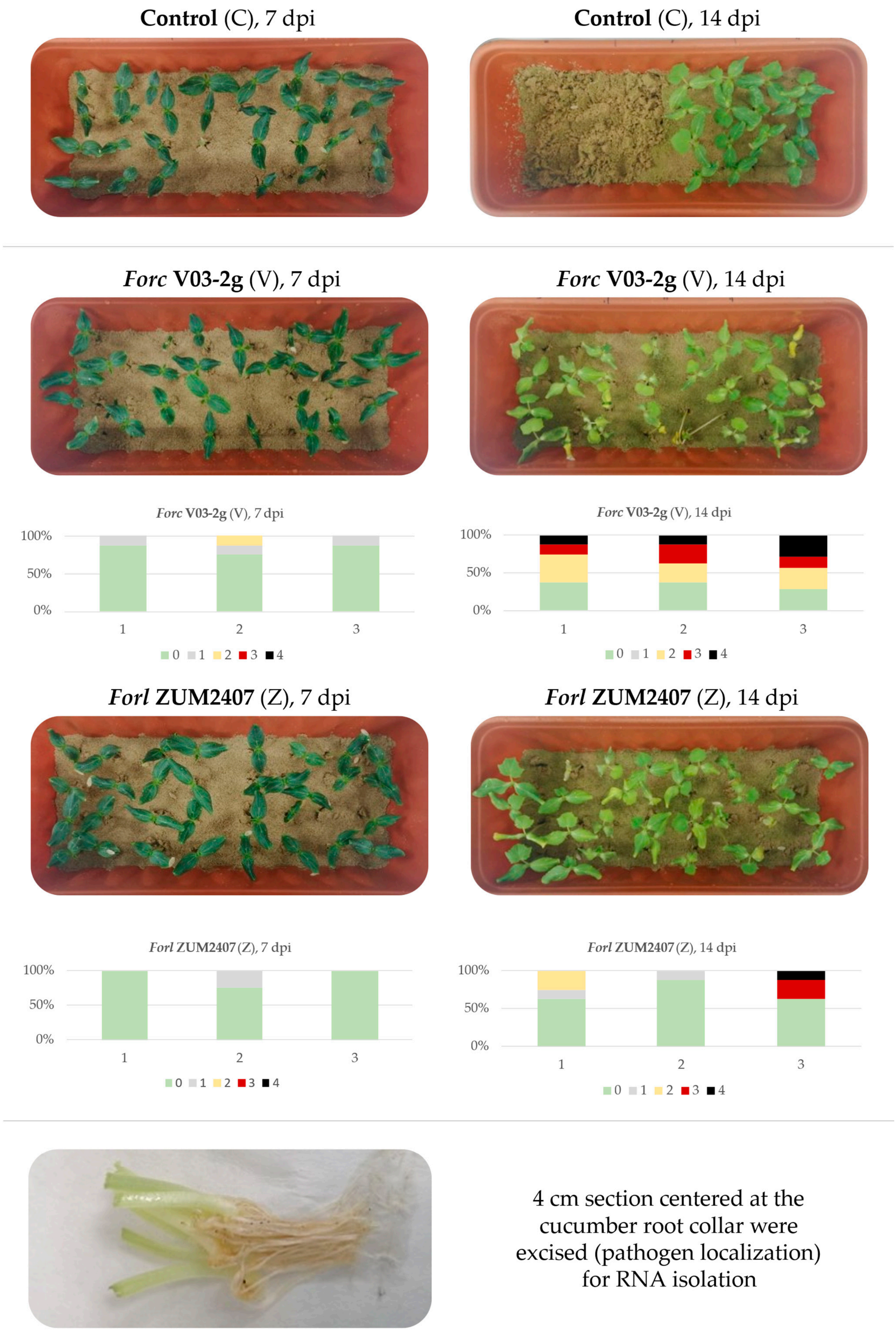

Figure S1. Cucumber samples at 7 and 14 dpi before RNA extraction and disease score for each three replicates.

Control (C), 2 dpi *Forc* **V03-2g** (V), 2 dpi *Forl* **ZUM2407** (Z), 2 dpi

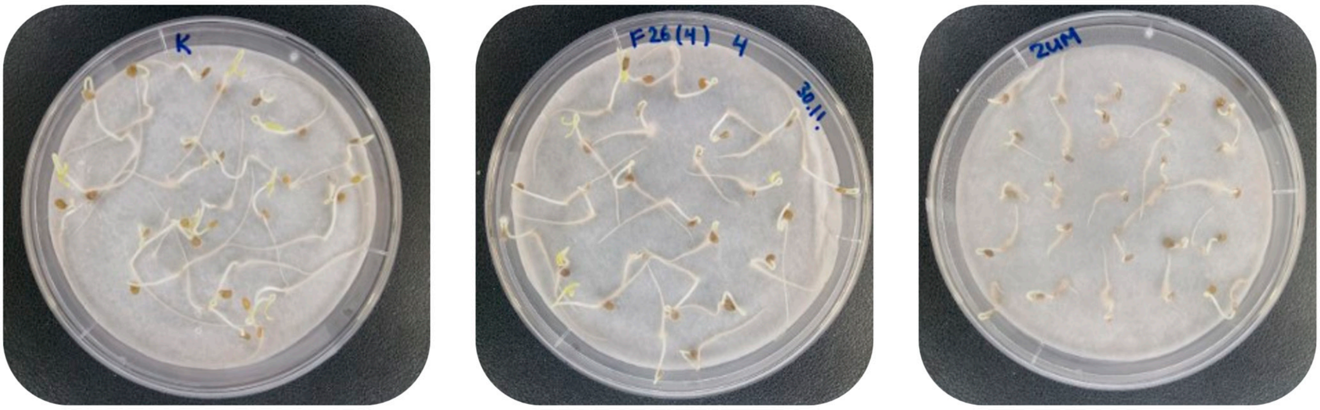

Figure S2. Tomato samples at 2 dpi before RNA extraction.

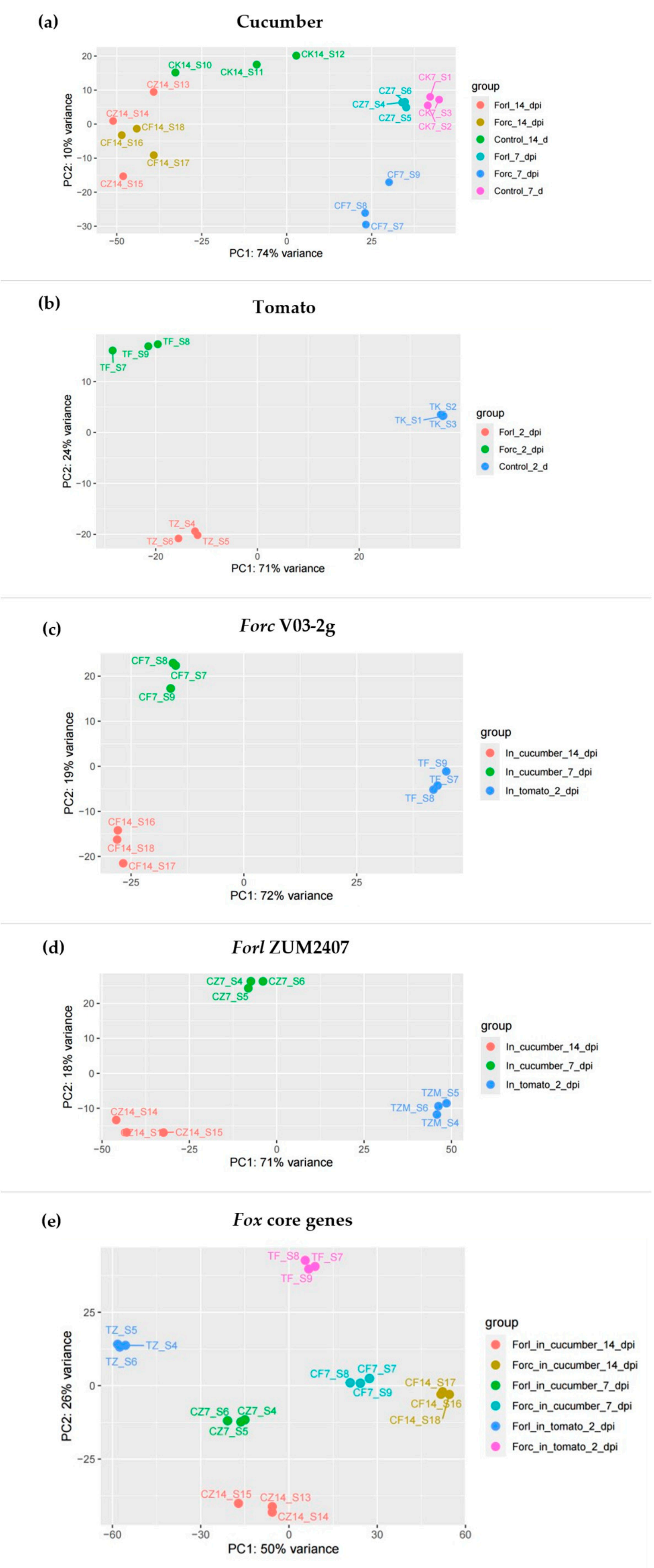

Figure S3. PCA-plots of (**a**) cucumber, (**b**) tomato, (**c**) *Forc* V03-2g, (**d**) *Forl* ZUM2407 and (**e**) *Fusarium oxysporum* core transcripts.

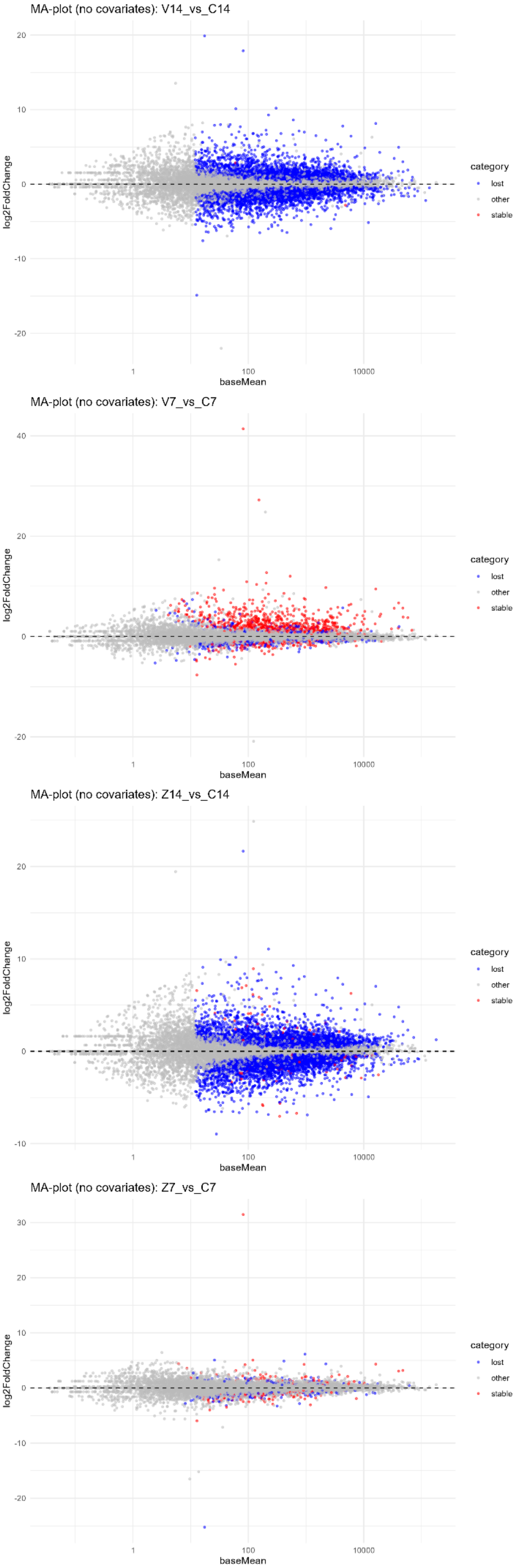

Figure S4a. MA-plots comparing differential expression in cucumber plants between models with and without fungal read percentage as a covariate. Both blue and red points represent genes that were differentially expressed in the model without the covariate. Blue points indicate genes that lost significance after including the covariate (lost), whereas red points indicate genes that remained significant (stable). Gray points represent non-significant genes.

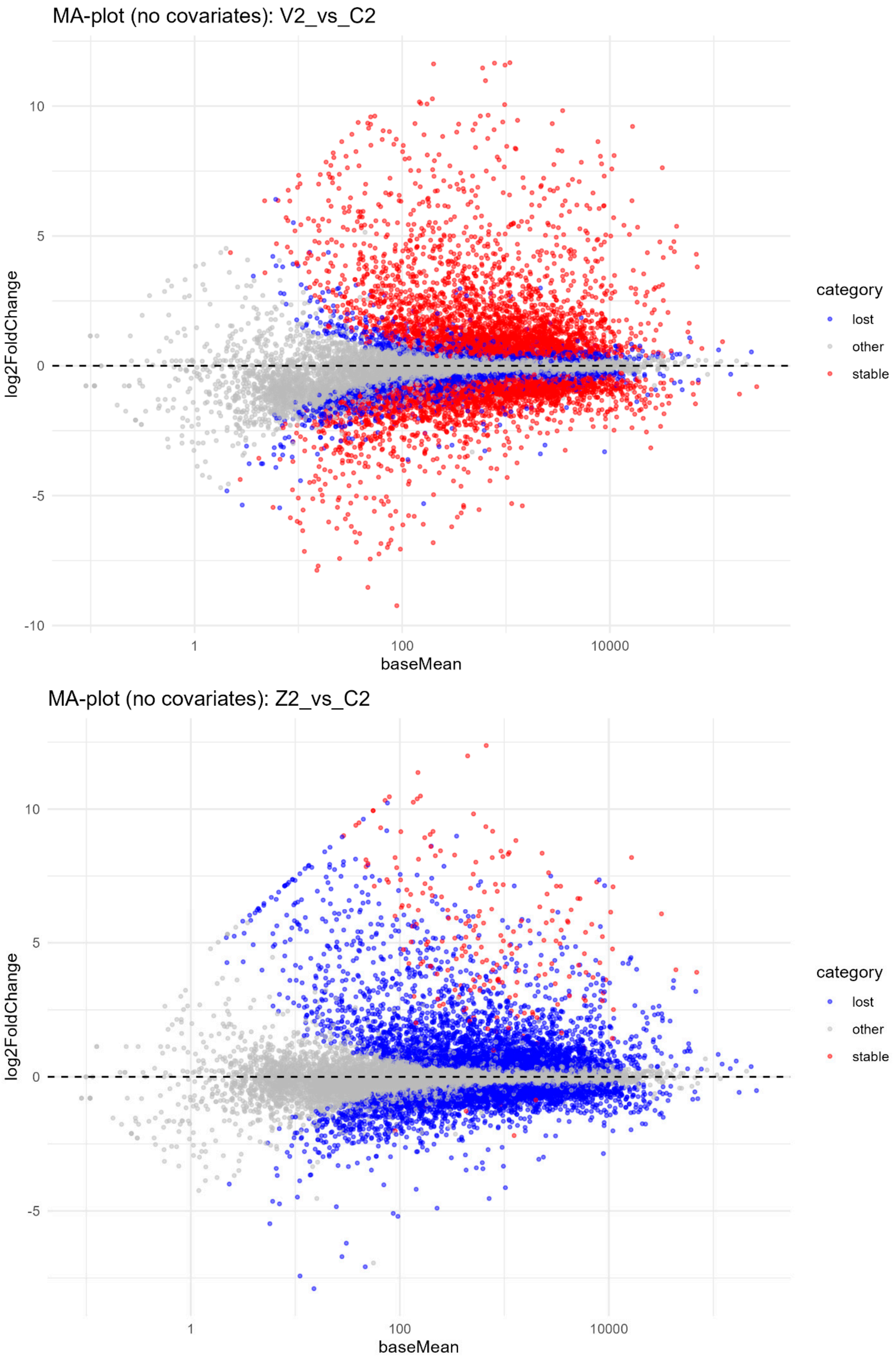

Figure S4b. MA-plots comparing differential expression in tomato plants between models with and without fungal read percentage as a covariate. Both blue and red points represent genes that were differentially expressed in the model without the covariate. Blue points indicate genes that lost significance after including the covariate (lost), whereas red points indicate genes that remained significant (stable). Gray points represent non-significant genes.

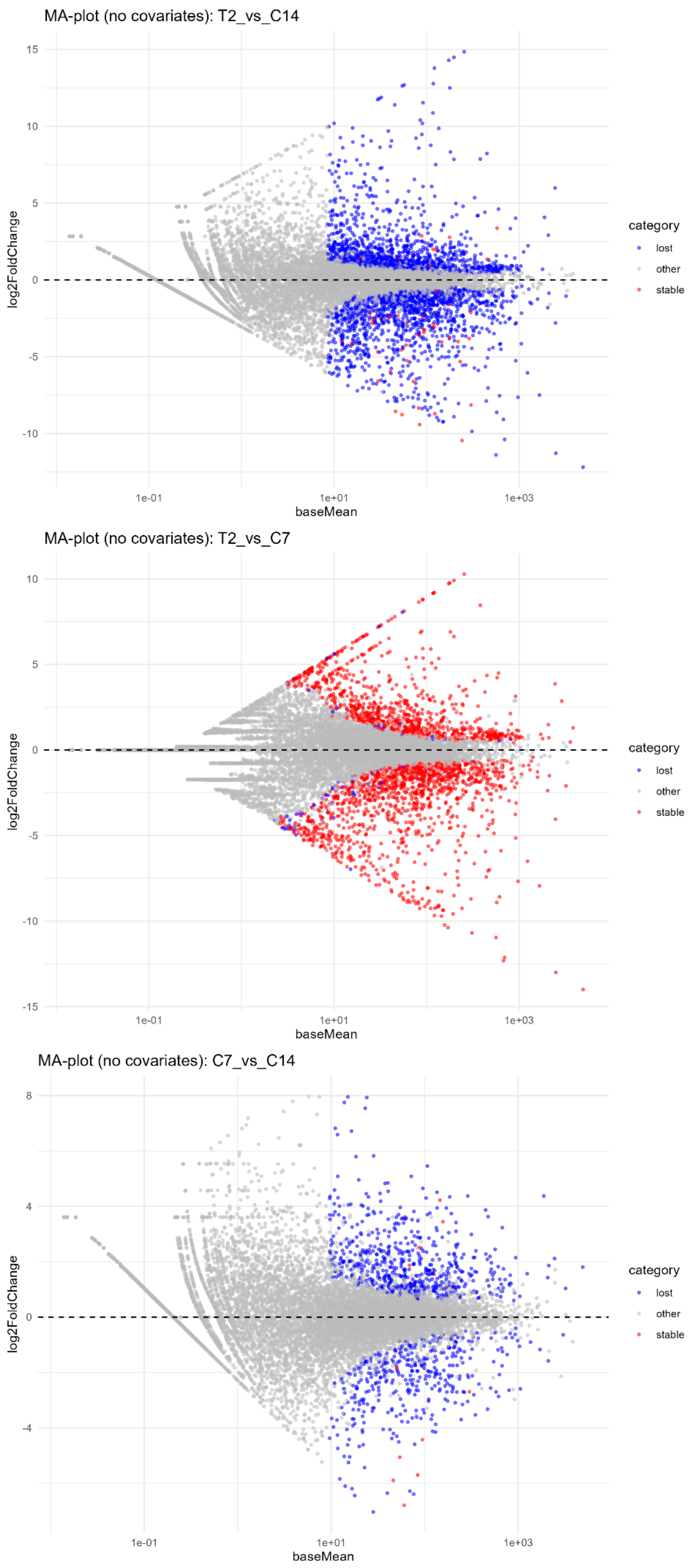

Figure S4c. MA-plots comparing differential expression in *Forc* V03-2g between models with and without fungal read percentage as a covariate. Both blue and red points represent genes that were differentially expressed in the model without the covariate. Blue points indicate genes that lost significance after including the covariate (lost), whereas red points indicate genes that remained significant (stable). Gray points represent non-significant genes.

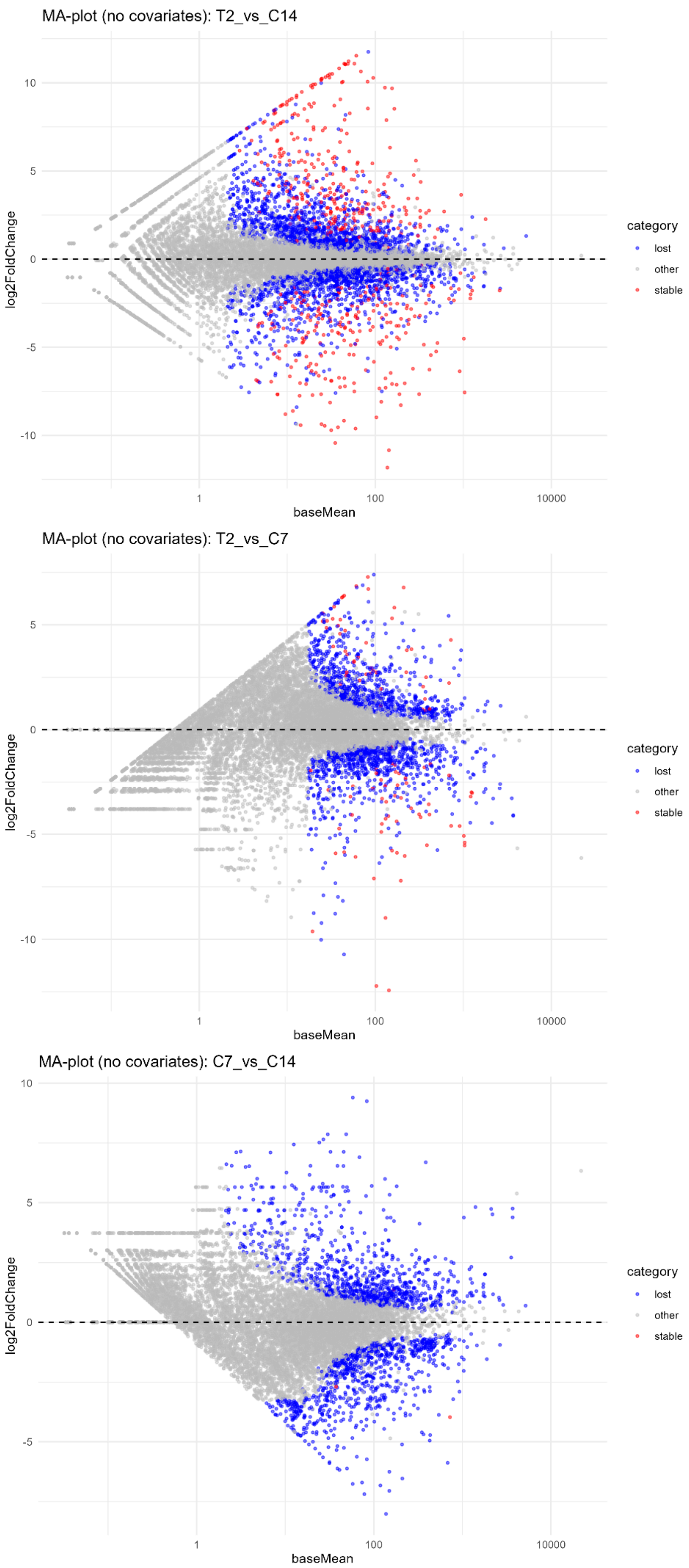

Figure S4d. MA-plots comparing differential expression in *Forl* ZUM2407 between models with and without fungal read percentage as a covariate. Both blue and red points represent genes that were differentially expressed in the model without the covariate. Blue points indicate genes that lost significance after including the covariate (lost), whereas red points indicate genes that remained significant (stable). Gray points represent non-significant genes.

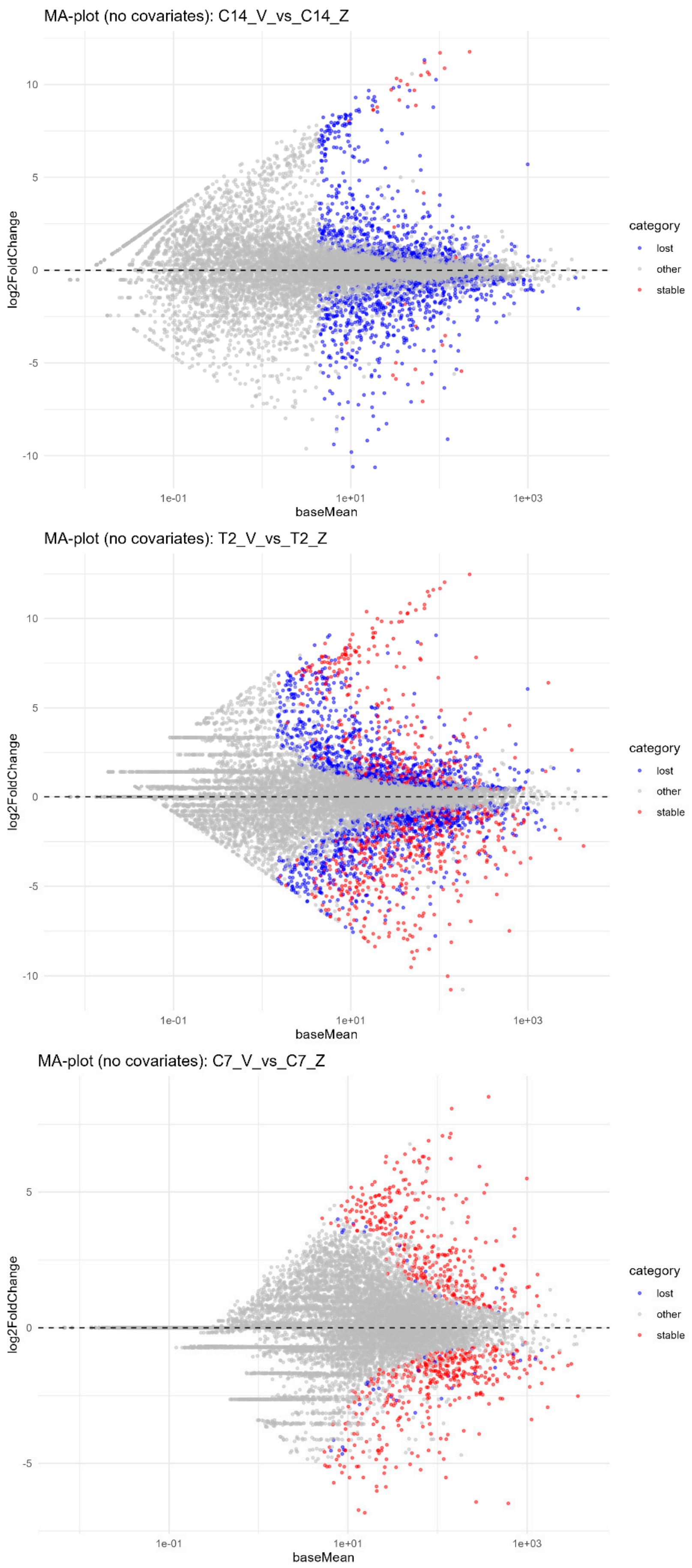

Figure S4e. MA-plots comparing differential expression in *Fusarium oxysporum* core genes between models with and without fungal read percentage as a covariate. Both blue and red points represent genes that were differentially expressed in the model without the covariate. Blue points indicate genes that lost significance after including the covariate (lost), whereas red points indicate genes that remained significant (stable). Gray points represent non-significant genes.

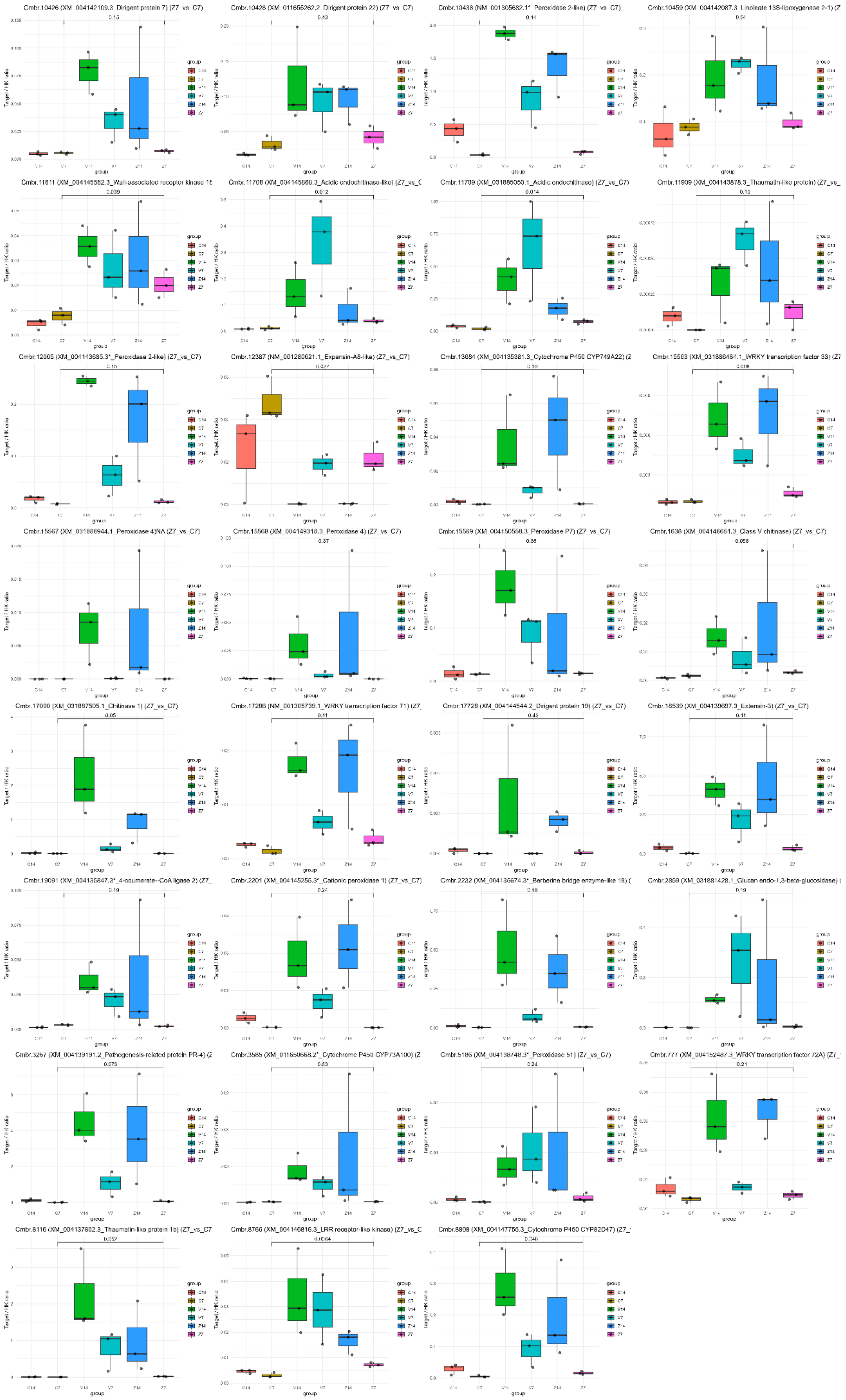

Figure S5a. Boxplots of relative expression (Target/HK ratio) for cucumber immune response genes (Table 1). DESeq2-normalized counts of target genes were divided by the geometric mean of DESeq2-normalized counts of three cucumber housekeeping (HK) genes (tubulin, actin, EF1-α). P-values (t-test) are shown for Z7 vs C7

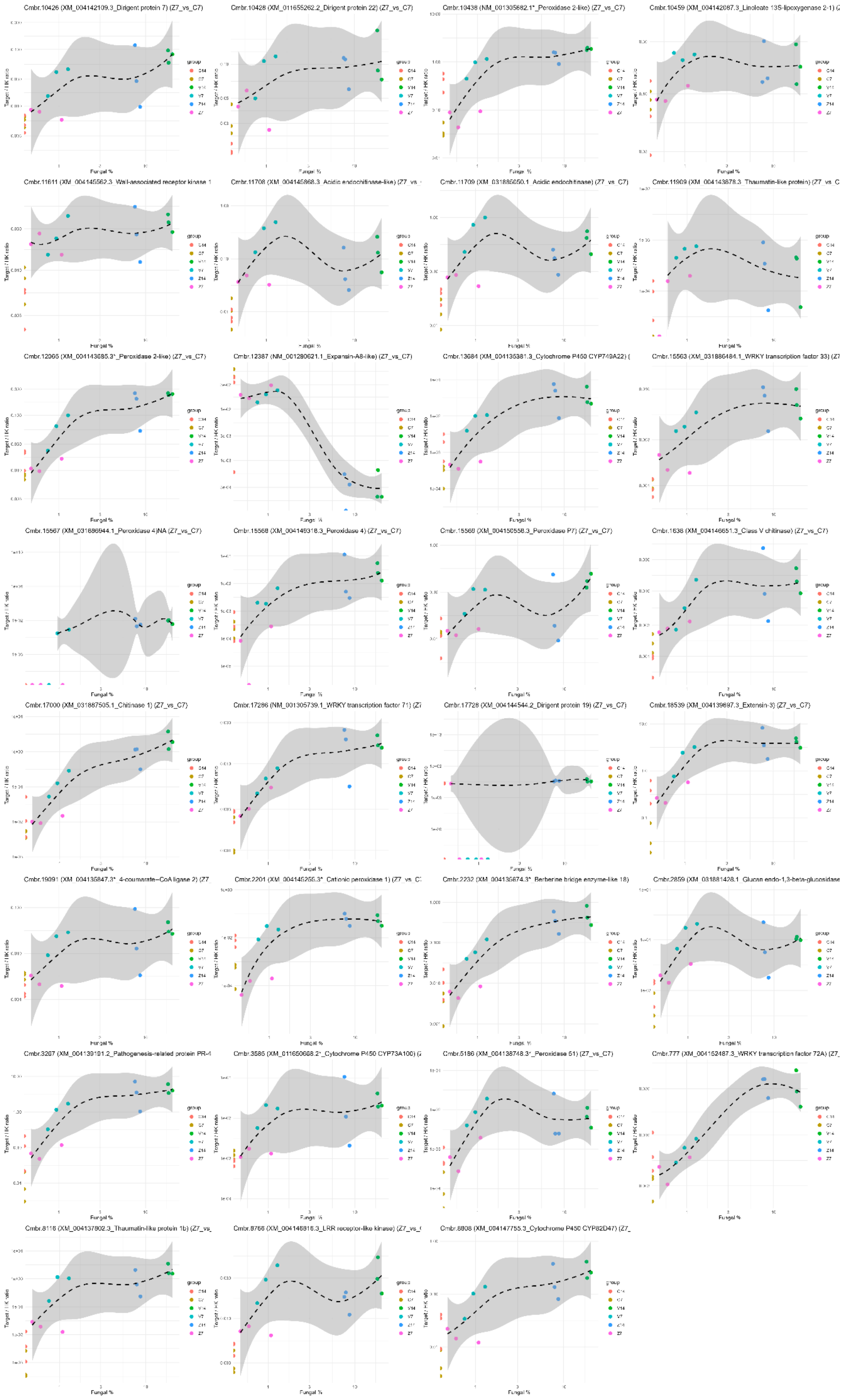

Figure S5b. Relationship between relative expression of individual cucumber defense genes listed in Table 1 (Target/HK ratio) and fungal read percentage (Fungal %).

(a) Cucumber response to*Forc* V03-2g at 7 dpi

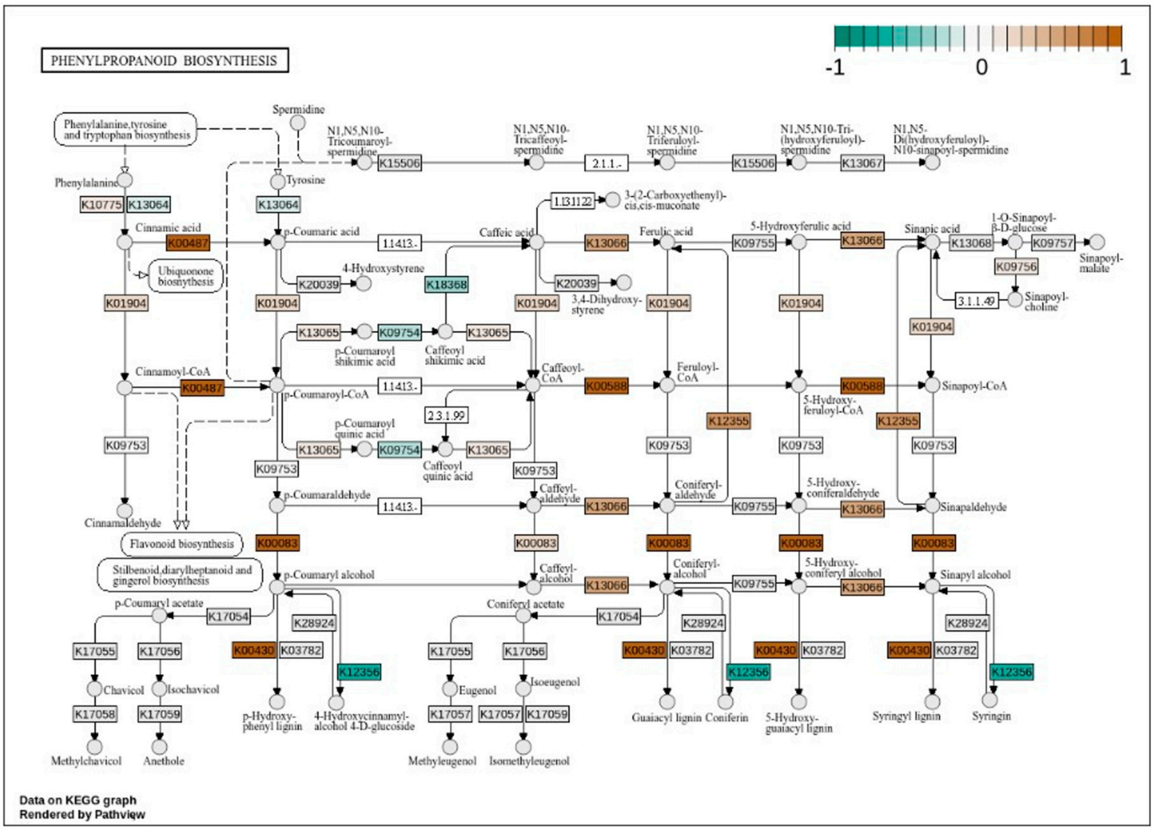

(b) Cucumber response to*Forl* ZUM2407 at 7 dpi

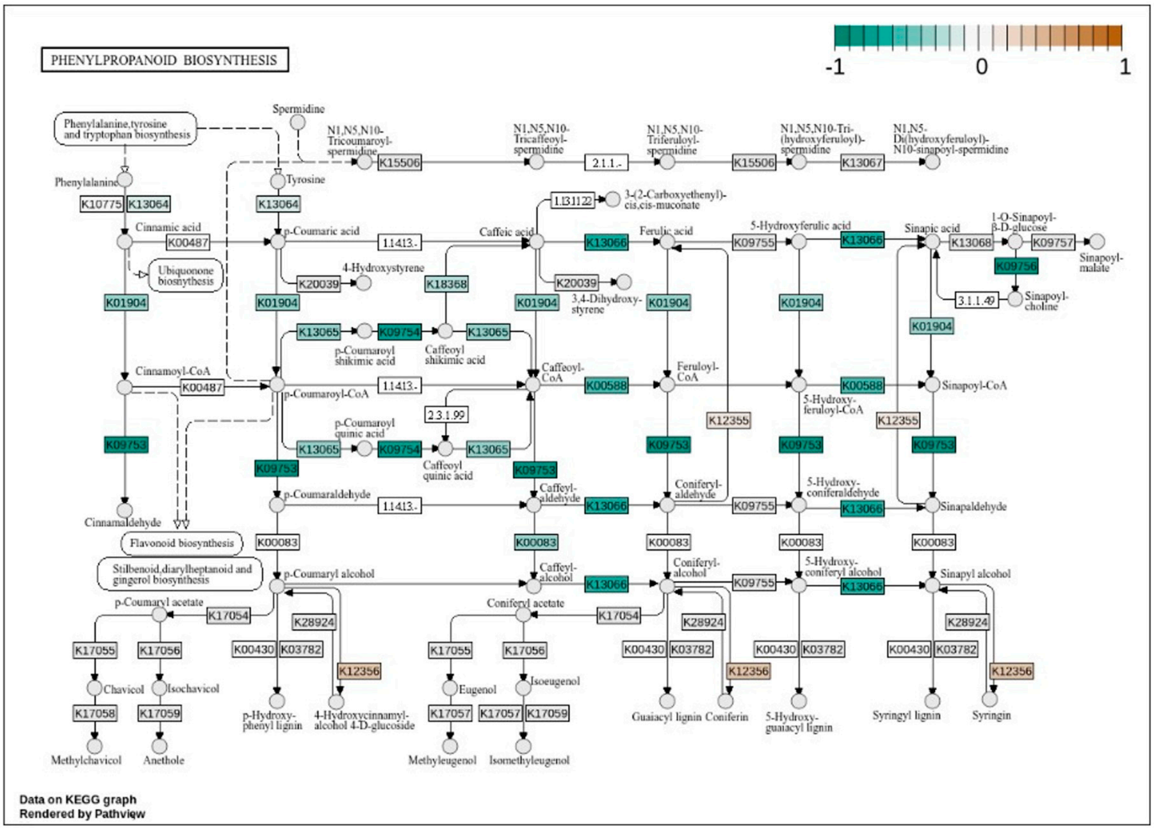

Figure S6. Phenylpropanoid biosynthesis (ko00940) in cucumber at 7 dpi, colored by mean log2 fold change of the host genes mapped to each KEGG ortholog node on a brown for induction and teal for repression scale. The pathway is induced during *Forc* V03-2g infection, while under *Forl* ZUM2407 infection it is repressed over the same nodes.

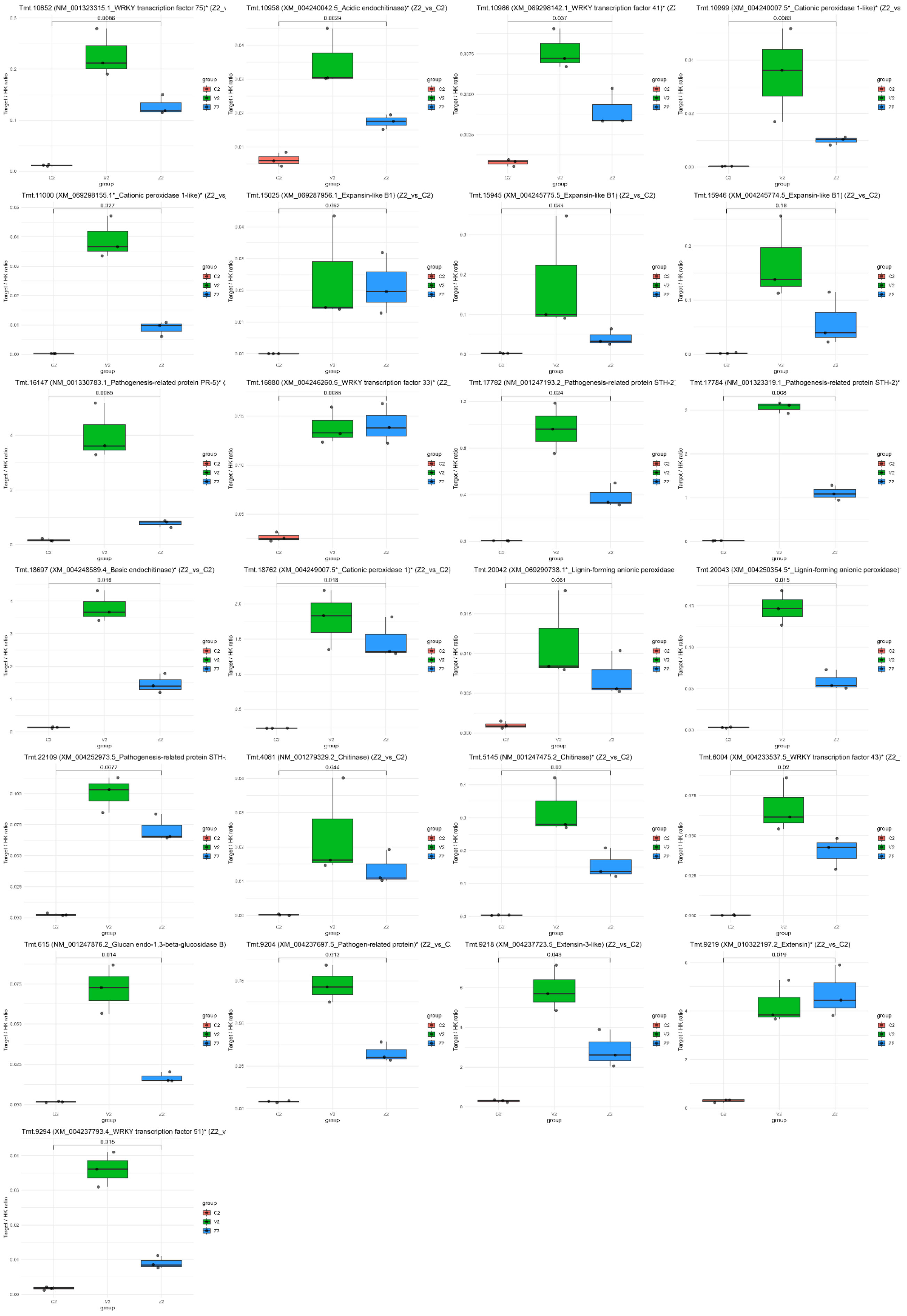

Figure S7a. Boxplots of relative expression (Target/HK ratio) for tomato immune response genes (Table 2). DESeq2-normalized counts of target genes were divided by the geometric mean of DESeq2-normalized counts of three tomato housekeeping (HK) genes (tubulin, actin, EF1-α). P-values (t-test) are shown for Z2 vs C2

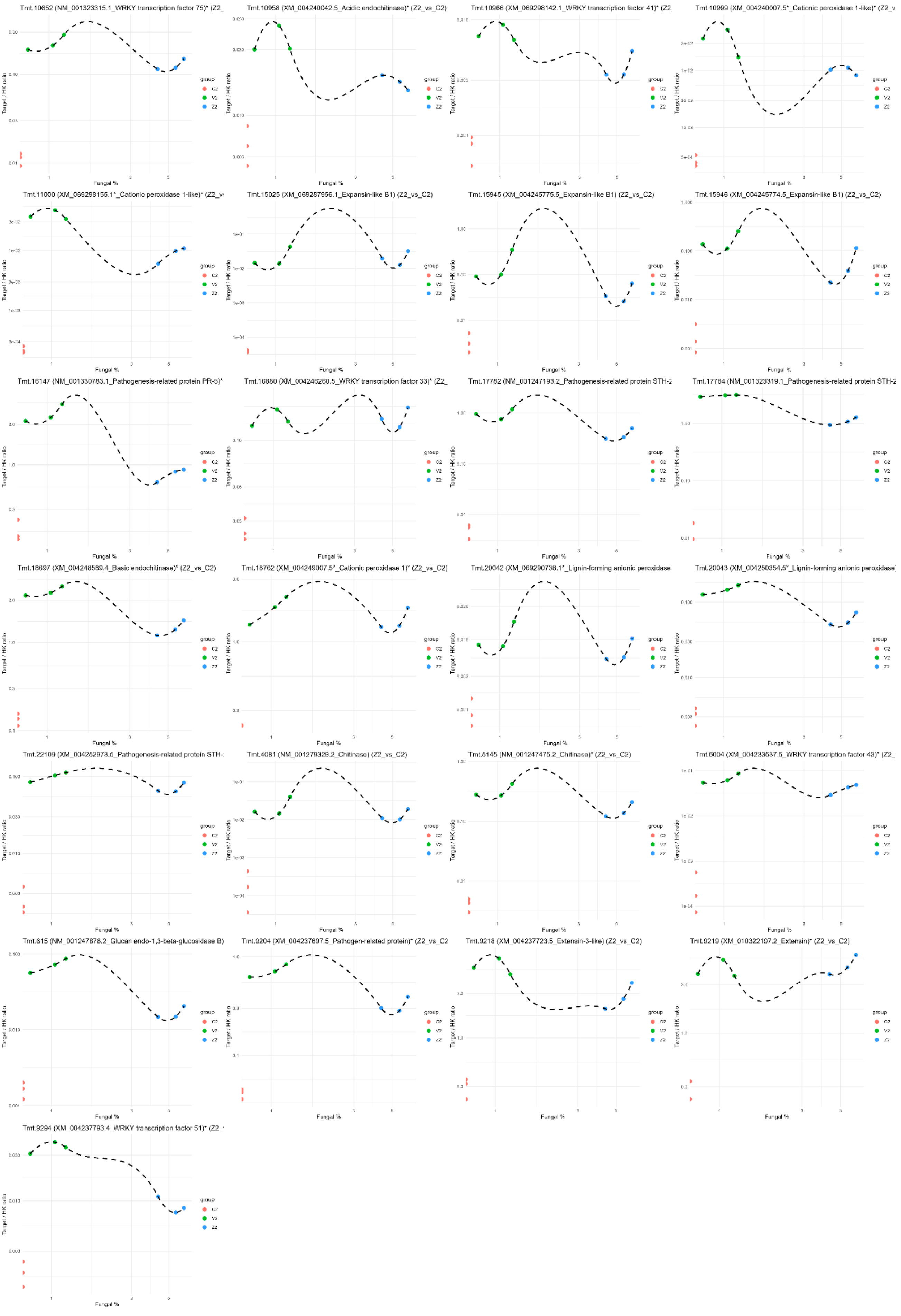

Figure S7b. Relationship between relative expression of individual tomato defense genes listed in Table 2 (Target/HK ratio) and fungal read percentage (Fungal %).

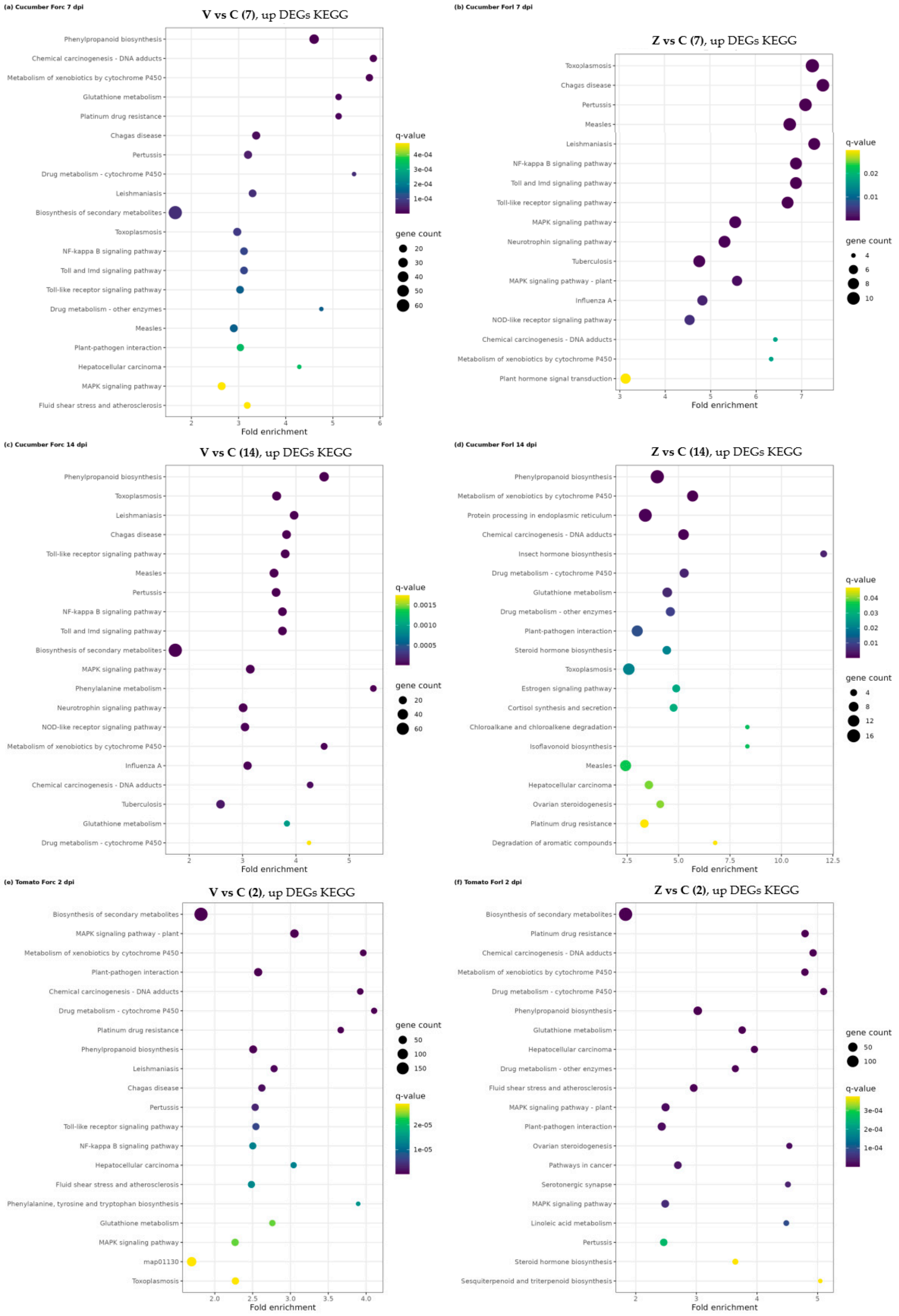

Figure S8. KEGG pathway over representation among genes induced during infection, for cucumber at 7 and 14 dpi and tomato at 2 dpi treated with *Forc* V03-2g and *Forl* ZUM2407. C – untreated healthy plants; V – plants pretreated with *Forc* V03-2g; Z – plants pretreated with *Forl* ZUM2407; numbers in brackets 7 and 14 indicate days post inoculation (dpi). Dot size denotes gene count and color denotes the adjusted p value. Panels: (**a**) V vs C at 7 dpi, (**b**) Z vs C at 7 dpi, (**c**) V vs C at 14 dpi, (**d**) Z vs C at 14 dpi, (**e**) V vs C at 2 dpi, (**f**) Z vs C at 2 dpi.

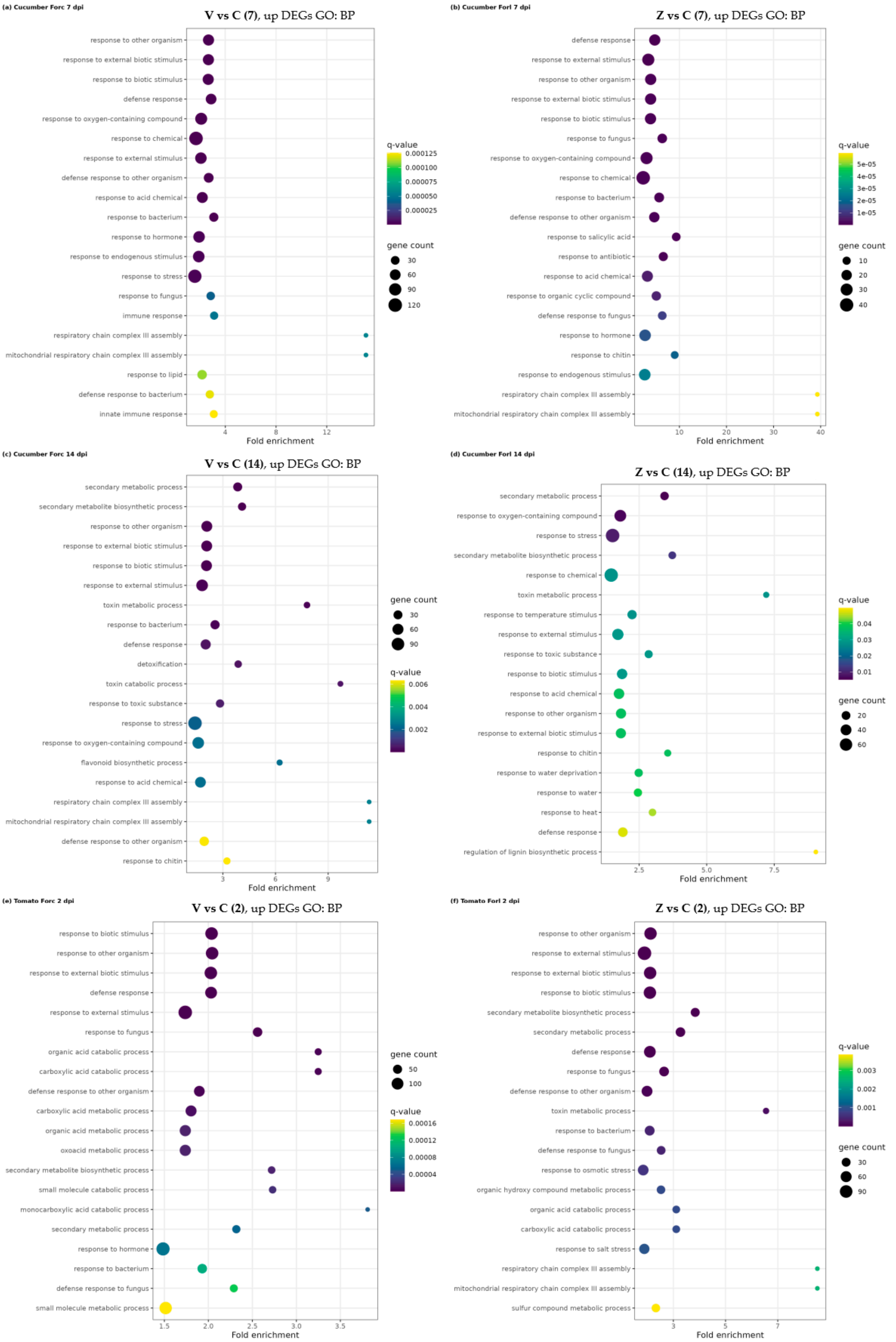

Figure S9. Gene Ontology (biological process) over representation among genes induced during infection, for the same six contrasts as Figure S8. Panels follow the same order, (a) to (f).

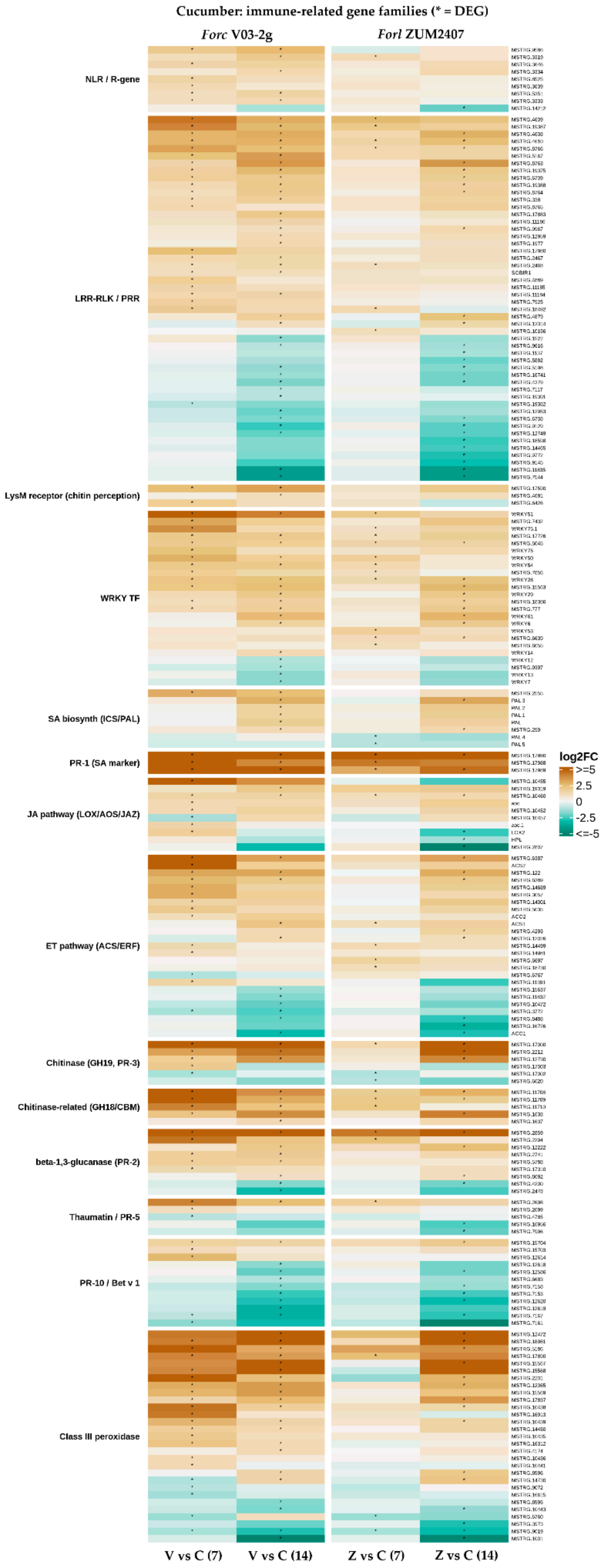

Figure S10. Expression of curated immune related gene families in cucumber during *Forc* V03-2g and *Forl* ZUM2407 infection, shown as log2 fold change relative to control. An asterisk marks a differentially expressed gene. C – untreated healthy plants; V – plants pretreated with *Forc* V03-2g; Z – plants pretreated with *Forl* ZUM2407; numbers in brackets 7 and 14 indicate days post inoculation (dpi).

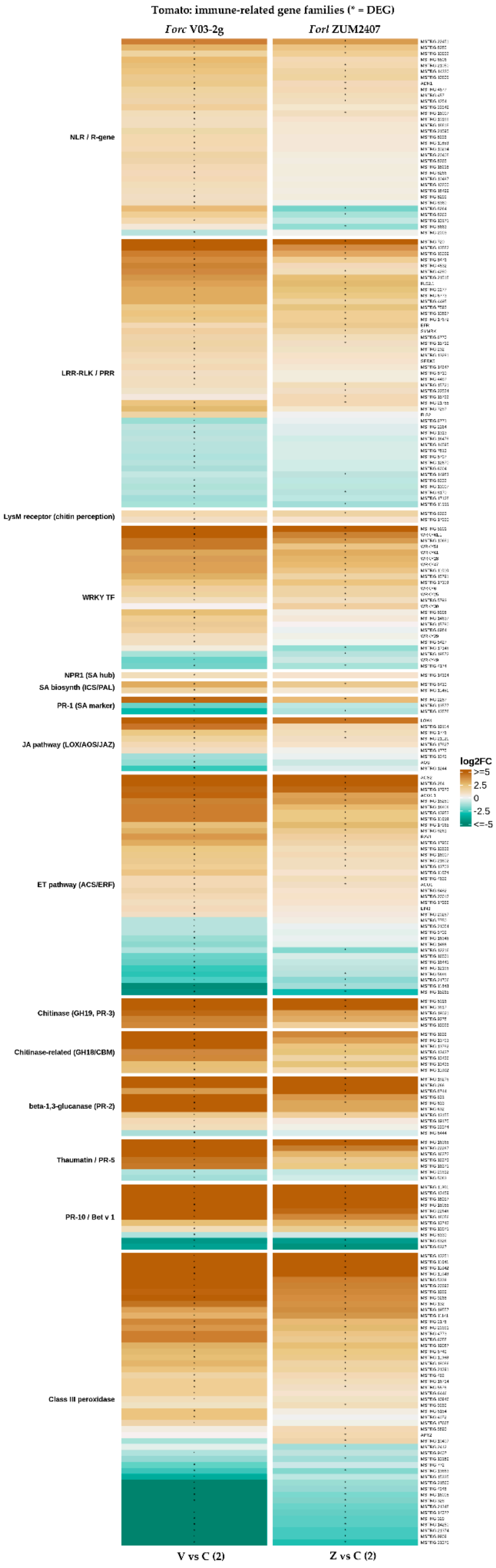

Figure S11. Expression of curated immune related gene families in tomato during *Forc* V03-2g and *Forl* ZUM2407 infection, shown as log2 fold change relative to control. An asterisk marks a differentially expressed gene. C – untreated healthy plants; V – plants pretreated with *Forc* V03-2g; Z – plants pretreated with *Forl* ZUM2407; numbers in brackets 2 indicate days post inoculation (dpi).

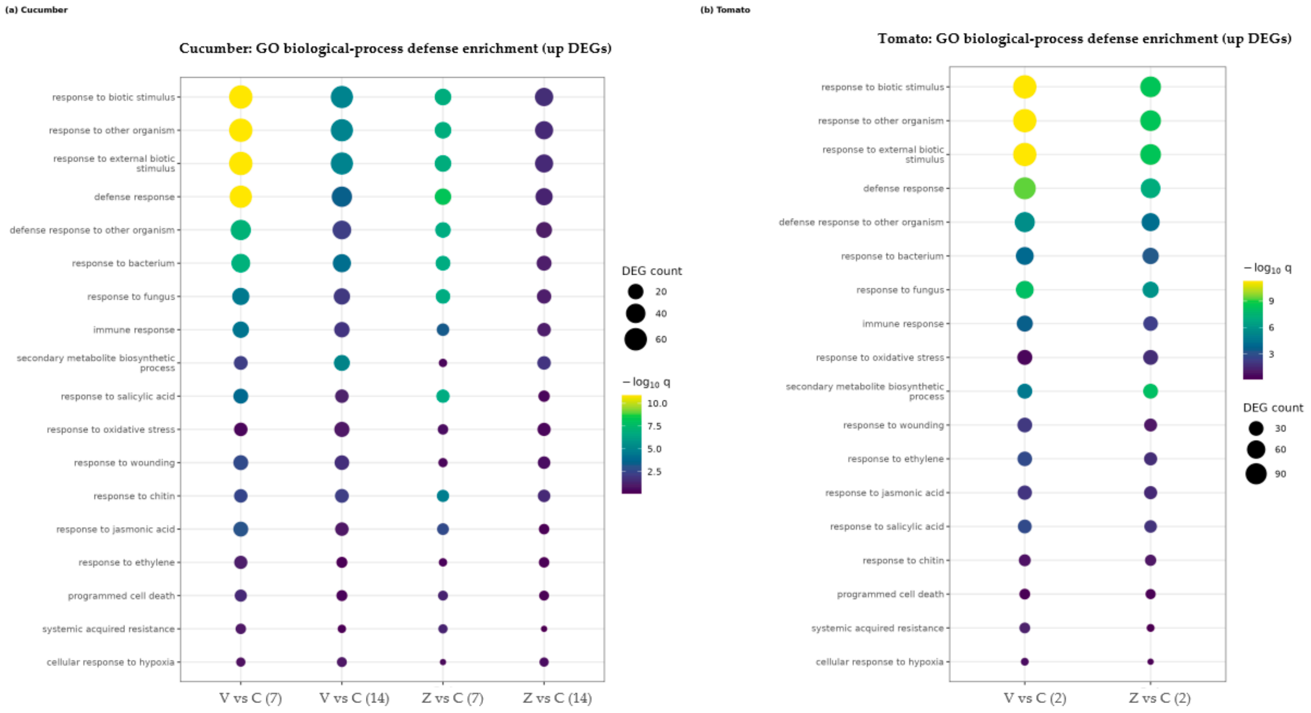

Figure S12. Comparative Gene Ontology (GO) enrichment among cucumber and tomato induced defense genes, (a) in cucumber at 7 and 14 dpi and (b) in tomato at 2 dpi.

Cucumber response to *Forc* V03-2g at 7 dpi | **Cucumber response to *Forl* ZUM2407 at 7 dpi**

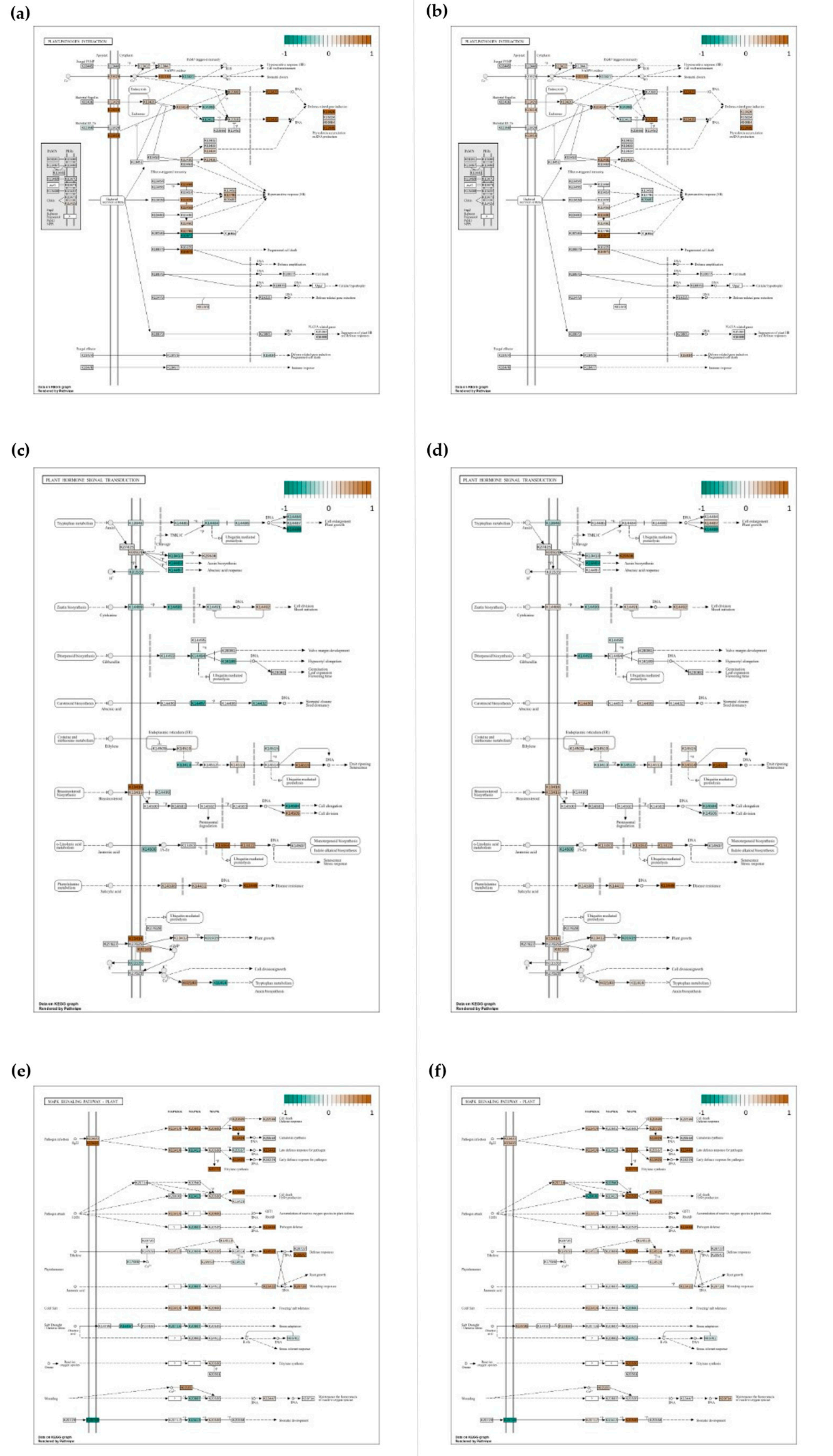

Figure S13. KEGG Pathview maps for the cucumber defense pathways, colored by mean log2 fold change per KEGG ortholog node on the brown for induction and teal for repression scale. Panels: plant pathogen interaction (a,b), plant hormone signal transduction (c,d) and MAPK signaling (e,f) in cucumber under *Forc* V03-2g (a,c,e) and *Forl* ZUM2407(b,d,f) infection at 7 dpi.

Tomato response to*Forc* V03-2g at 2 dpi	Tomato response to*Forl* ZUM2407 at 2 dpi

(a)

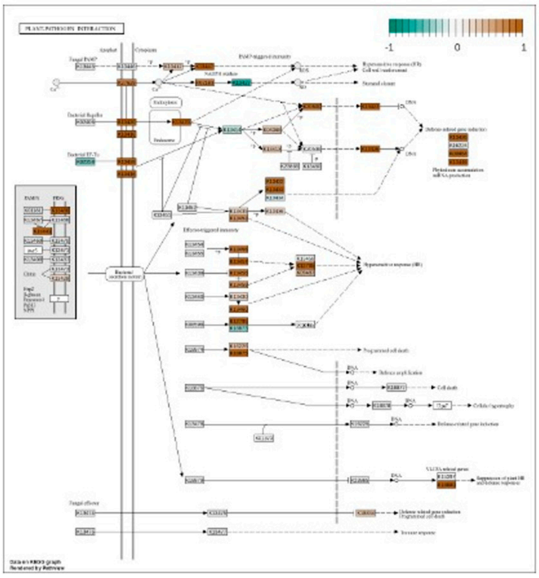

(b)

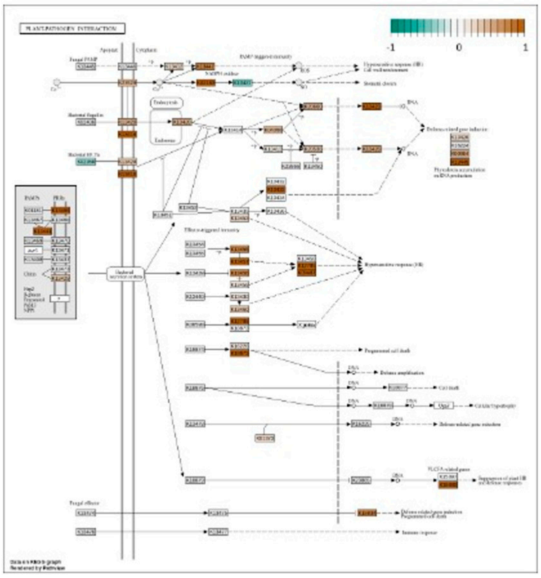

(c)

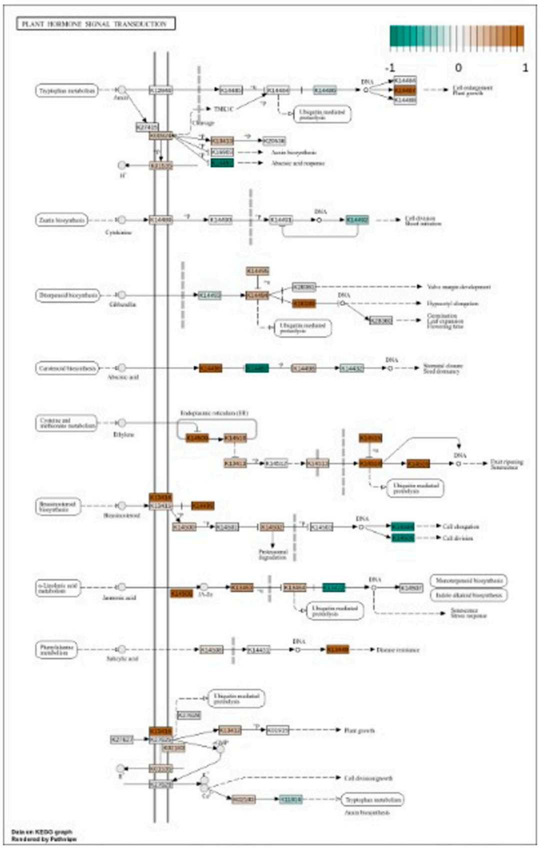

(d)

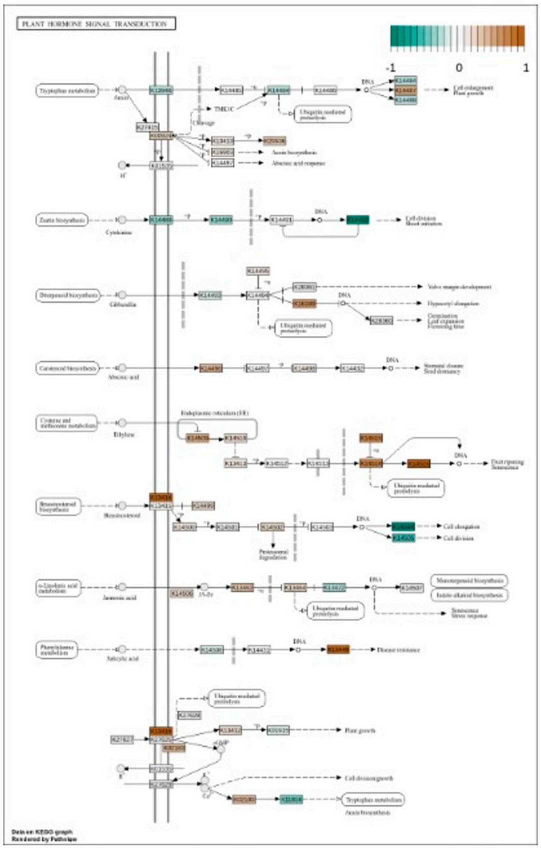

Figure S14. KEGG Pathview maps for the tomato defense pathways, colored by mean log2 fold change per KEGG ortholog node on the brown for induction and teal for repression scale. Panels: plant pathogen interaction (a,b), plant hormone signal transduction (c,d) in tomato under *Forc* V03-2g (a,c) and *Forl* ZUM2407 (b,d))at 2 dpi.

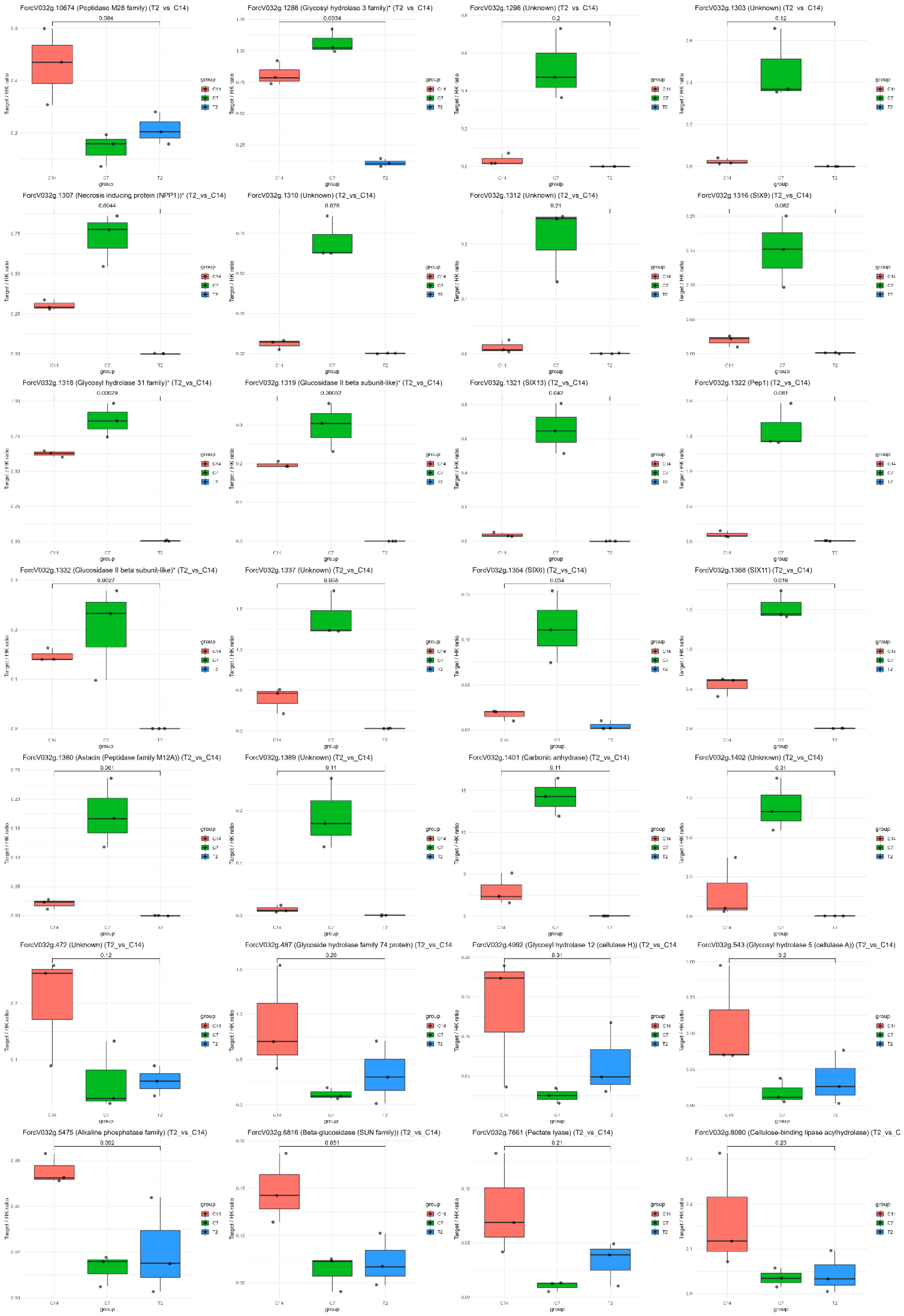

Figure S15a. Boxplots of relative expression (Target/HK ratio) for *Forc* V03-2g genes listed in Table 4. DESeq2-normalized counts of target genes were divided by the geometric mean of DESeq2-normalized counts of three *Forc* V03-2g housekeeping (HK) genes (tubulin, actin, TEF1-α). P-values (t-test) are shown for C14 vs T2

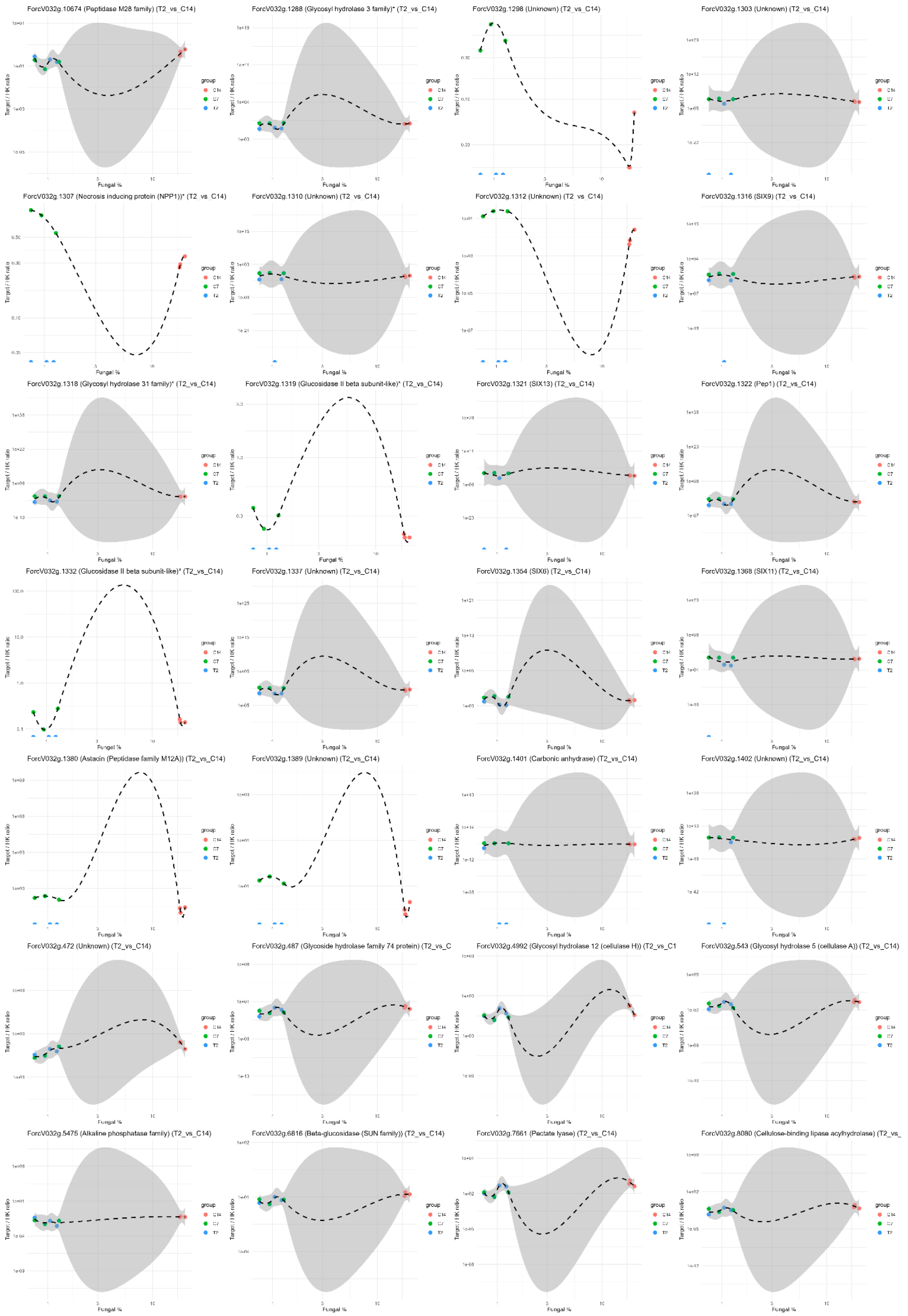

Figure S15b. Relationship between relative expression of individual *Forc* V03-2g genes listed in Table 4 (Target/HK ratio) and fungal read percentage (Fungal %).

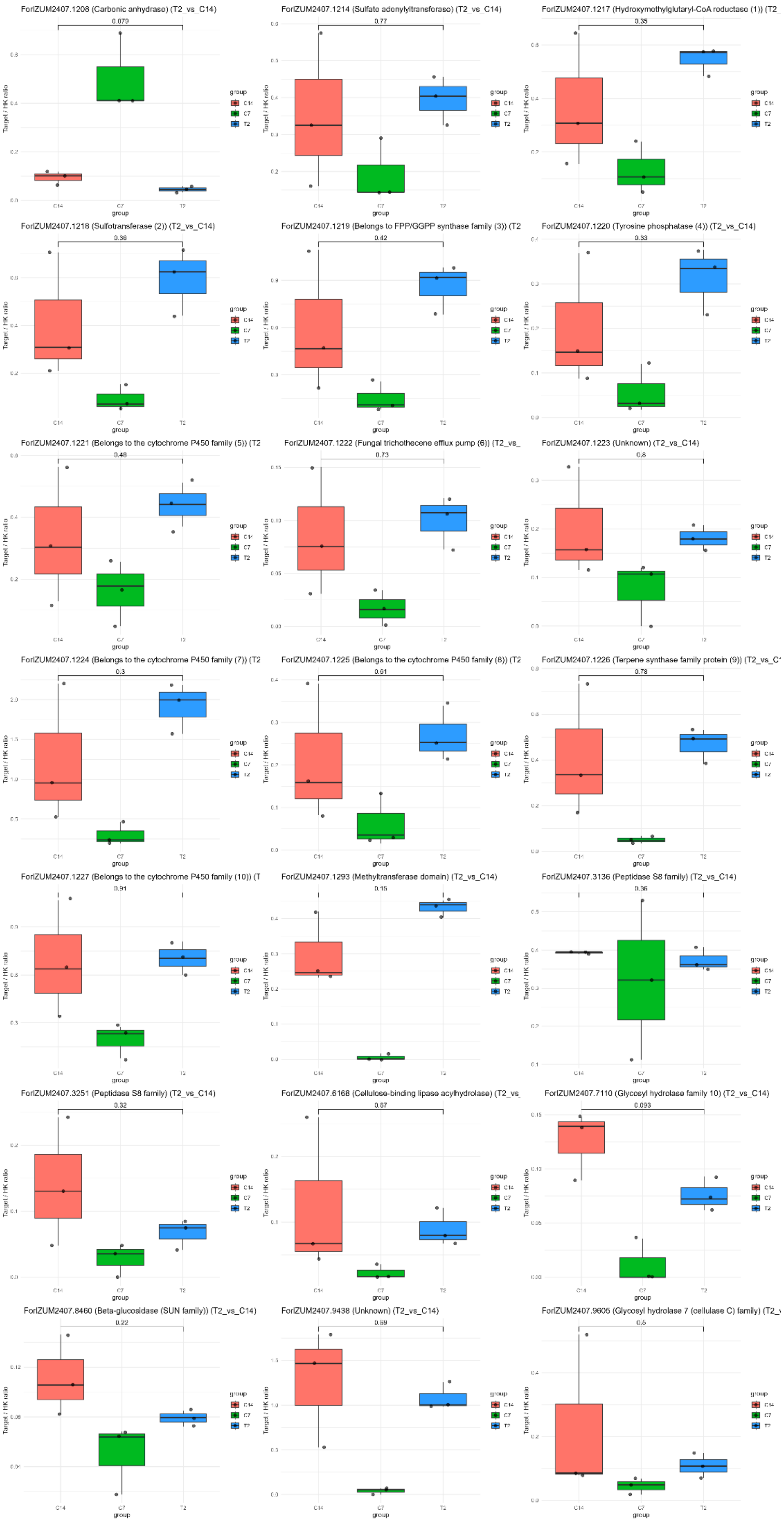

Figure S16a. Boxplots of relative expression (Target/HK ratio) for *Forl* ZUM2407 genes listed in Table 5. DESeq2-normalized counts of target genes were divided by the geometric mean of DESeq2-normalized counts of three *Forl* ZUM2407 housekeeping (HK) genes (tubulin, actin, TEF1-α). P-values (t-test) are shown for T2 vs C14.

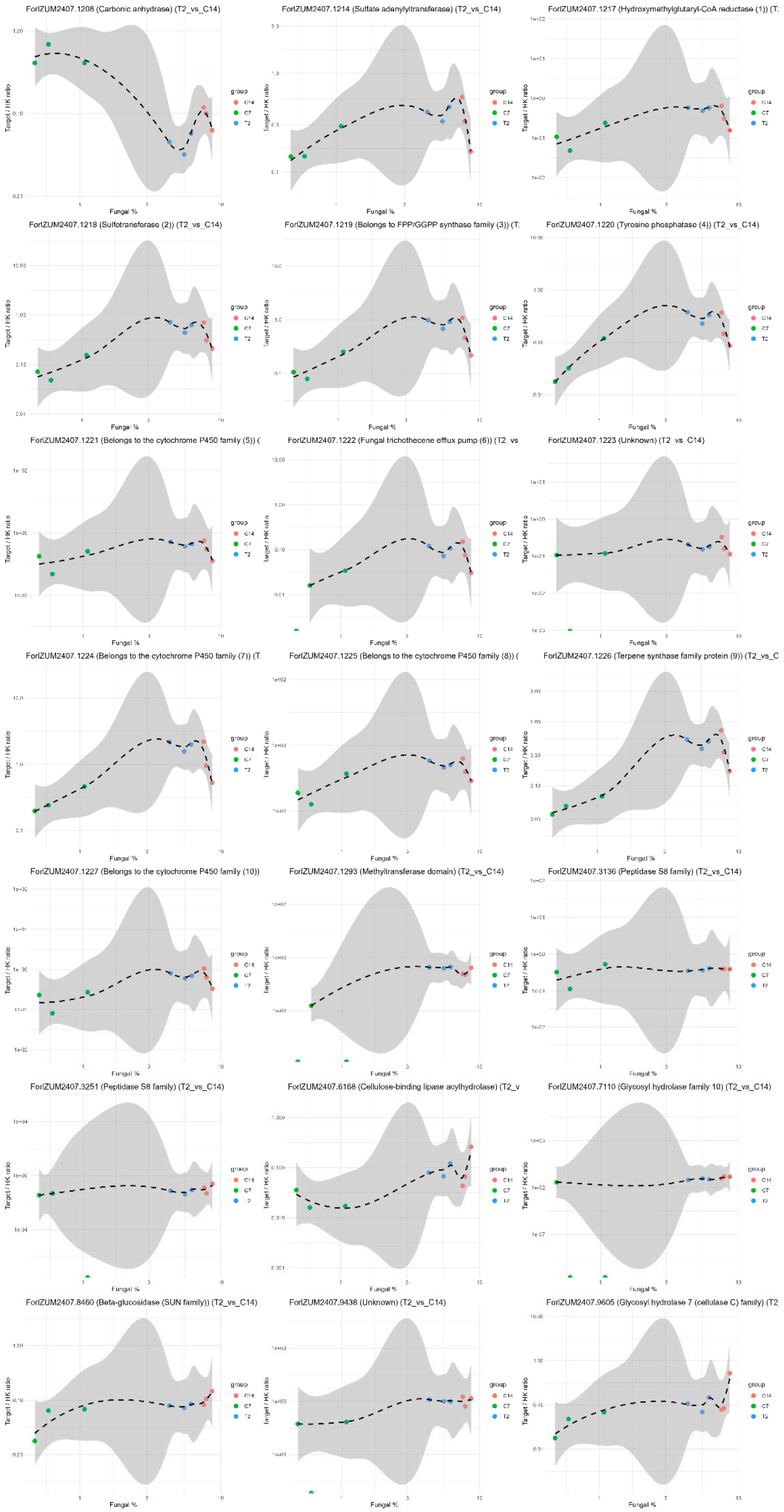

Figure S16b. Relationship between relative expression of individual *ForI* ZUM2407 genes listed in Table 5 (Target/HK ratio) and fungal read percentage (Fungal %).

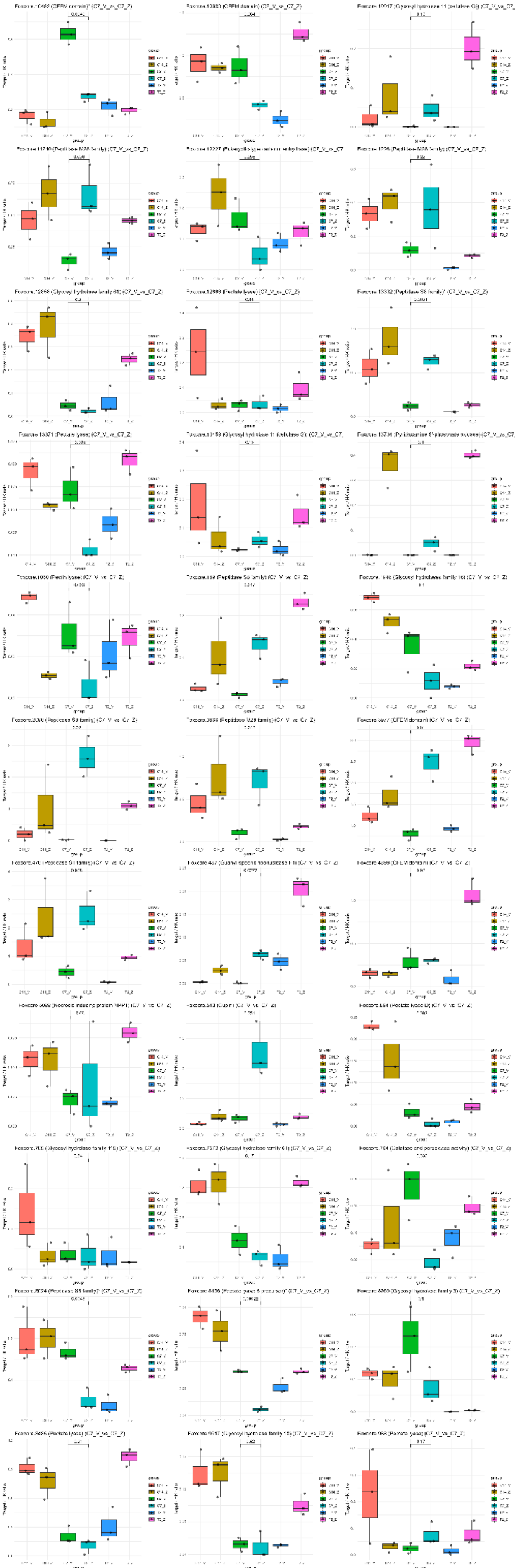

Figure S17a. Boxplots of relative expression (Target/HK ratio) for *Fusarium oxysporum* core secreted genes listed in Table 7. DESeq2-normalized counts of target genes were divided by the geometric mean of DESeq2-normalized counts of three *Fusarium oxysporum* housekeeping (HK) genes (tubulin, actin, TEF1-α). P-values (t-test) are shown for V7 vs Z7.

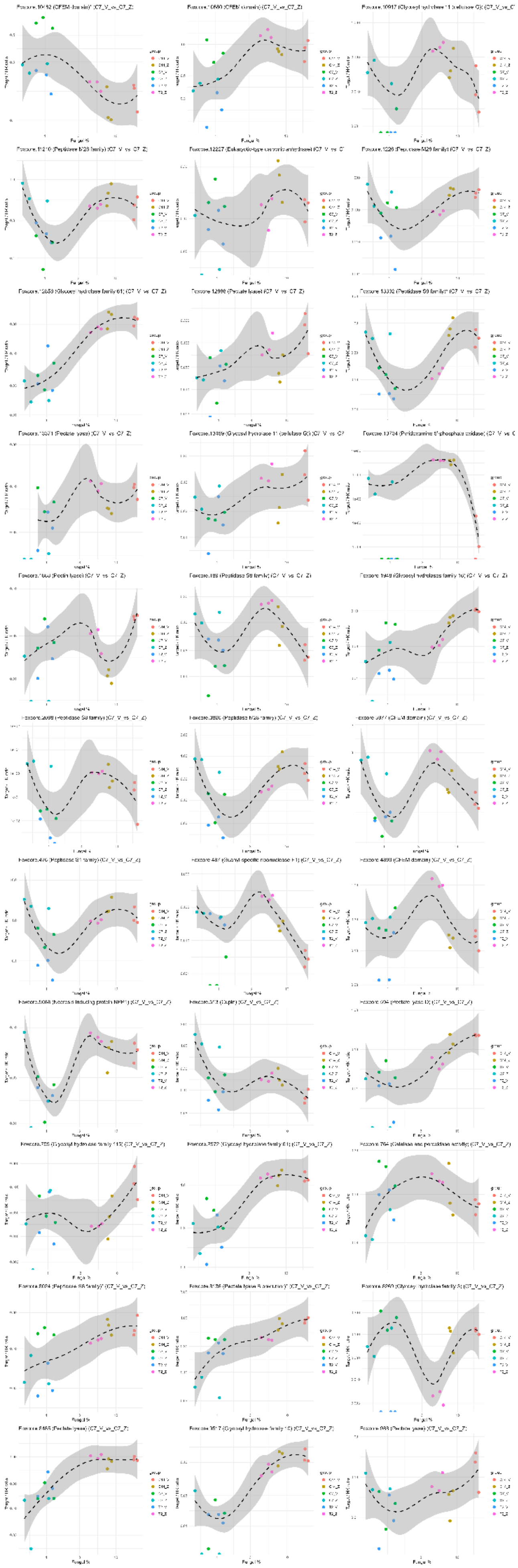

Figure S17b. Relationship between relative expression of individual *Fusarium oxysporum* core secreted genes listed in Table 7 (Target/HK ratio) and fungal read percentage (Fungal %).

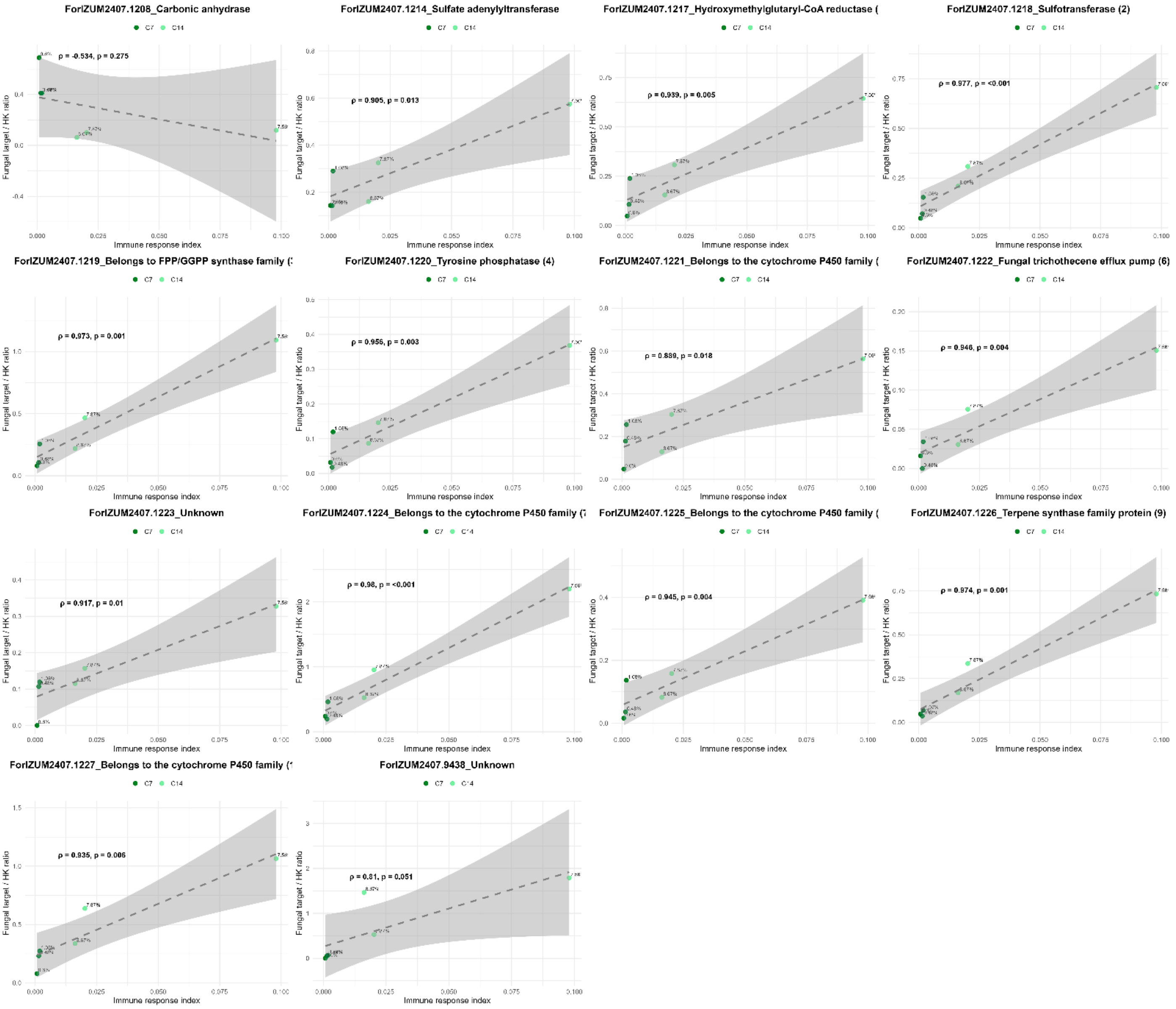

Figure S18a. Correlations between cucumber immune response index and relative expression of individual *ForI* ZUM2407 genes located on accessory genome. The immune index was calculated as the geometric mean of all cucumber genes from Table 1 (geometric mean of Target/HK ratios). Fungal gene expression also represented as Target/HK ratio (relation to geometric mean of three housekeeping (HK) genes (tubulin, actin, TEF1-α). Each panel represents one fungal gene. Points are colored by group (C7, C14), and fungal read percentages are shown. Pearson correlation coefficients (ϱ) and p-values are indicated.

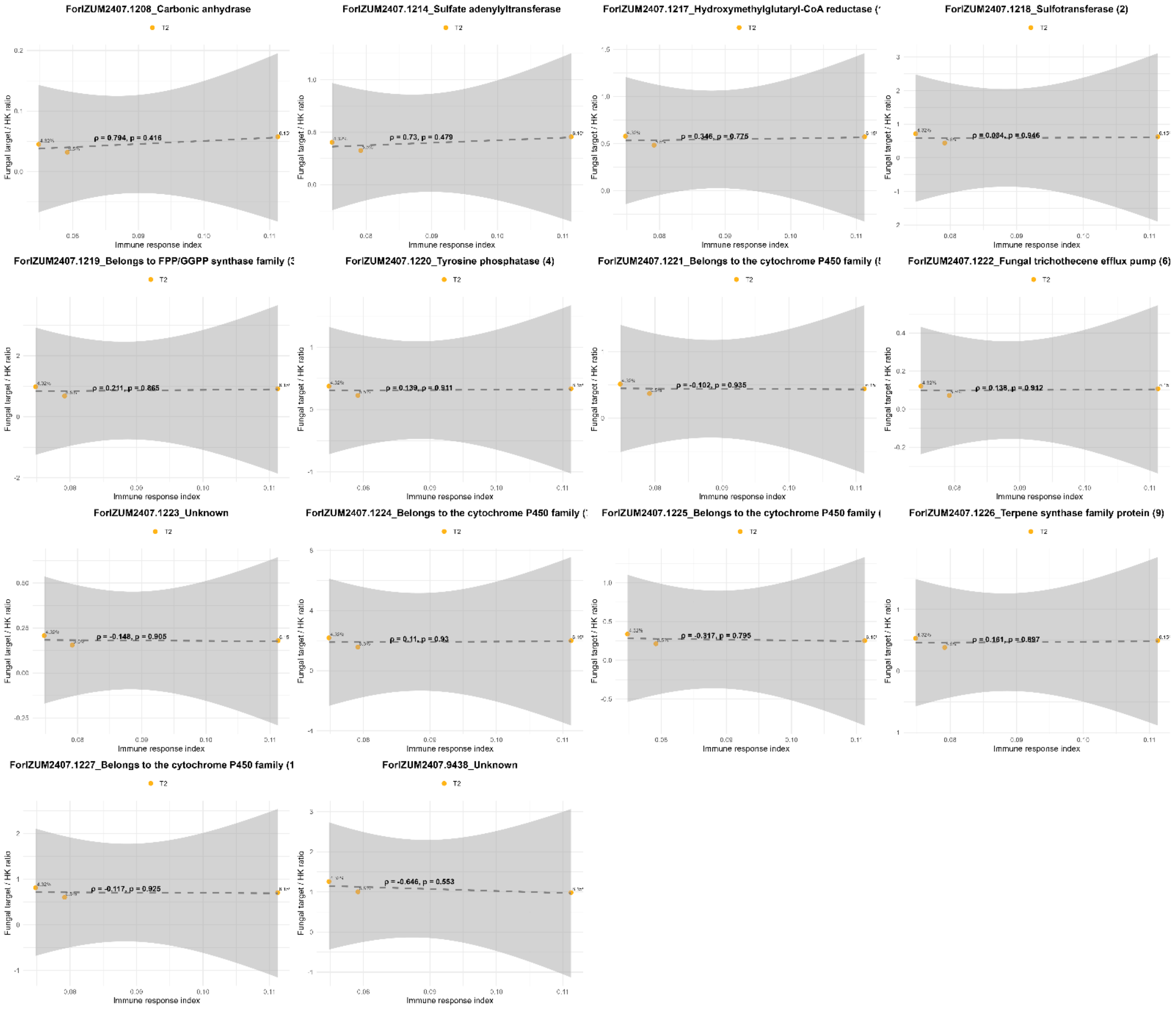

Figure S18b. Correlations between tomato immune response index and relative expression of individual *Forl* ZUM2407 genes located on accessory genome. The immune index was calculated as the geometric mean of all tomato genes from Table 2 (geometric mean of Target/HK ratios). Fungal gene expression also represented as Target/HK ratio (relation to geometric mean of three housekeeping (HK) genes (tubulin, actin, TEF1-α). Each panel represents one fungal gene. Points are colored by group (T2), and fungal read percentages are shown. Pearson correlation coefficients (ϱ) and p-values are indicated.

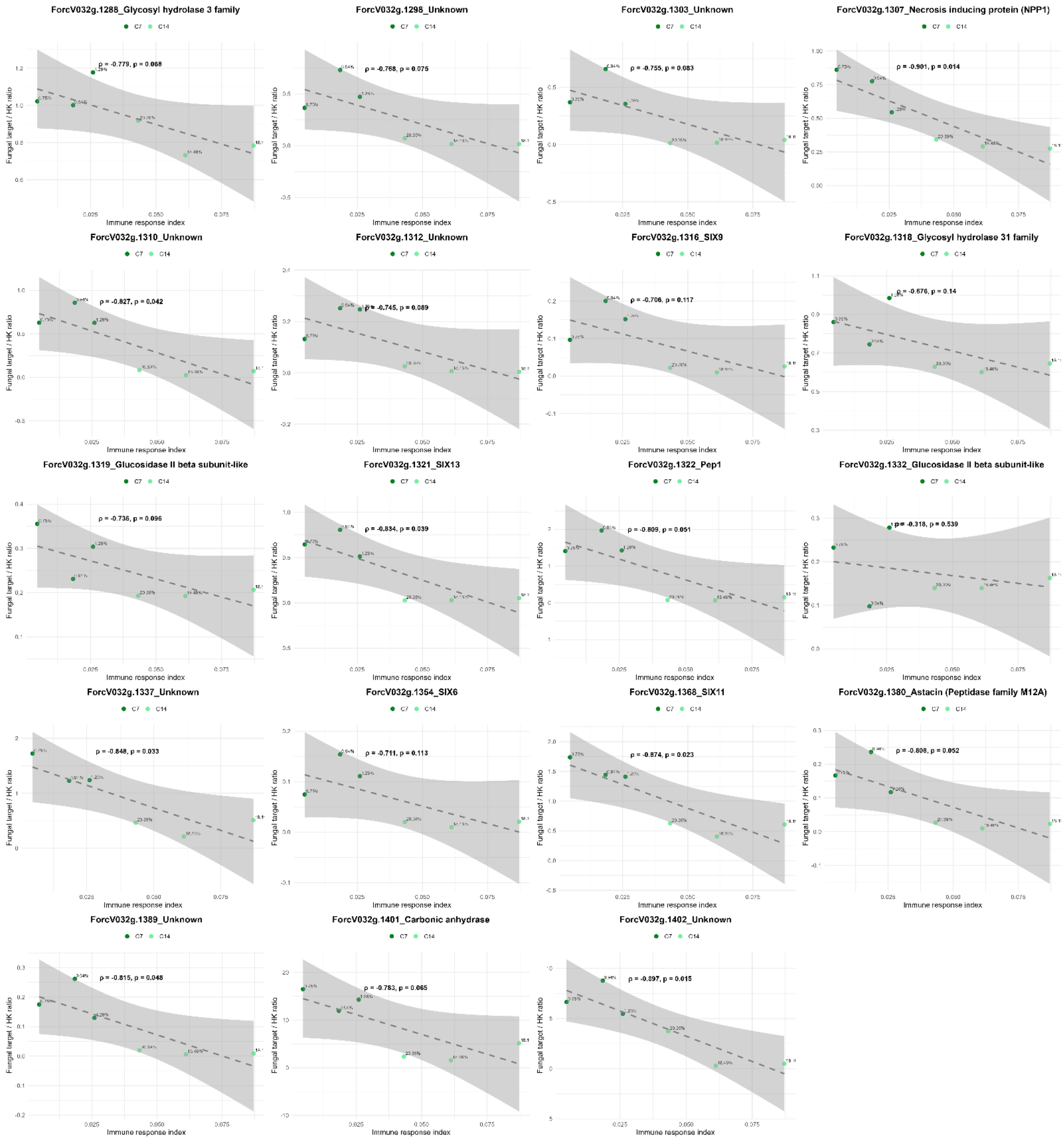

Figure 19a. Correlations between cucumber immune response index and relative expression of individual *Forc* V03-2g genes located on accessory genome. The immune index was calculated as the geometric mean of all cucumber genes from Table 1 (geometric mean of Target/HK ratios). Fungal gene expression also represented as Target/HK ratio (relation to geometric mean of three housekeeping (HK) genes (tubulin, actin, TEF1-α). Each panel represents one fungal gene. Points are colored by group (C7, C14), and fungal read percentages are shown. Pearson correlation coefficients (ϱ) and p-values are indicated.

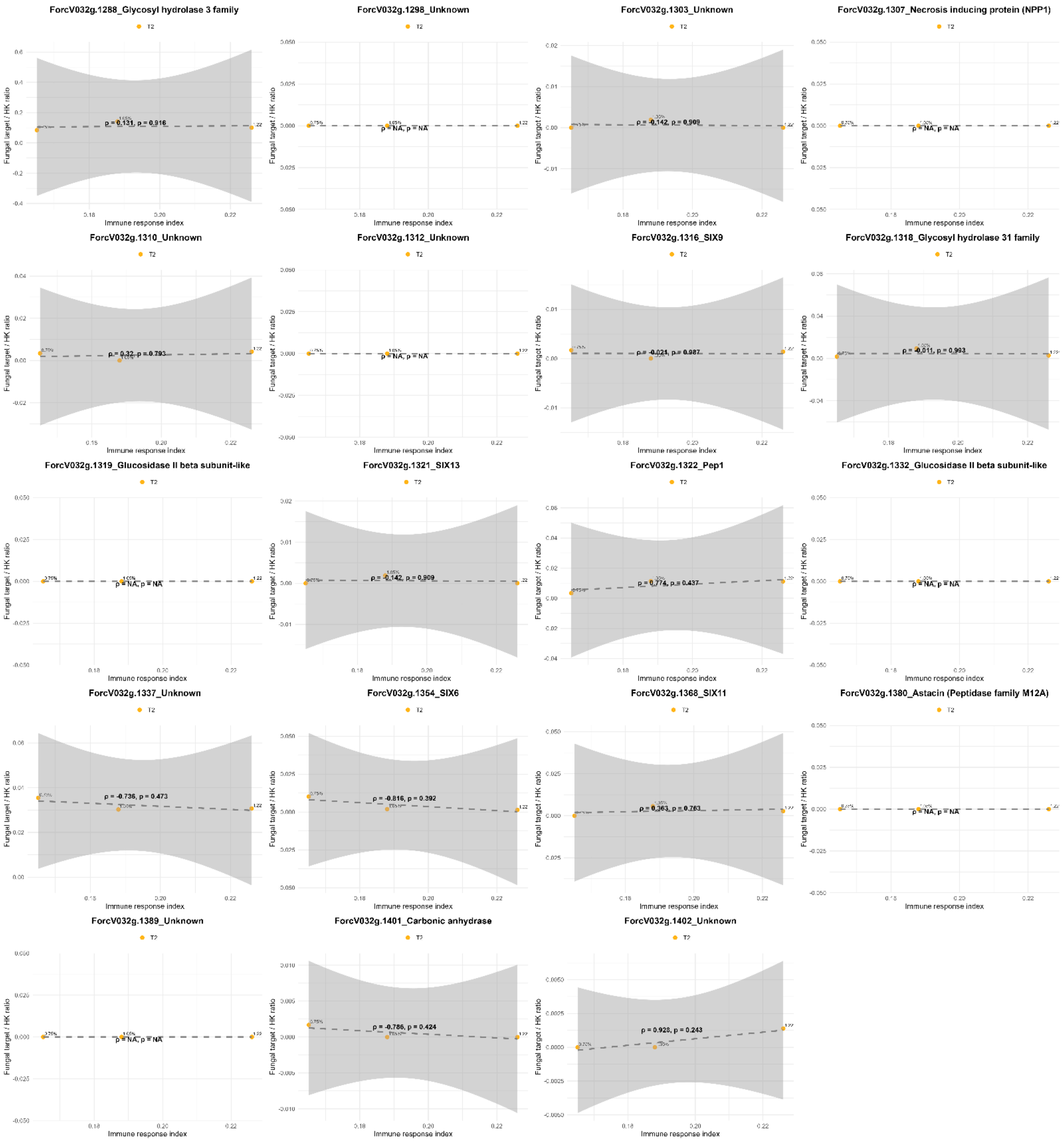

Figure 19b. Correlations between tomato immune response index and relative expression of individual *Forc* V03-2g genes located on accessory genome. The immune index was calculated as the geometric mean of all tomato genes from Table 2 (geometric mean of Target/HK ratios). Fungal gene expression also represented as Target/HK ratio (relation to geometric mean of three housekeeping (HK) genes (tubulin, actin, TEF1-α). Each panel represents one fungal gene. Points are colored by group (T2), and fungal read percentages are shown. Pearson correlation coefficients (ϱ) and p-values are indicated.
